# Identification of Ligand Binding Sites of Proteins Using the Gaussian Network Model

**DOI:** 10.1371/journal.pone.0016474

**Published:** 2011-01-25

**Authors:** Ceren Tuzmen, Burak Erman

**Affiliations:** Center for Computational Biology and Bioinformatics, Koc University, Istanbul Turkey; Koç University, Turkey

## Abstract

The nonlocal nature of the protein-ligand binding problem is investigated via the Gaussian Network Model with which the residues lying along interaction pathways in a protein and the residues at the binding site are predicted. The predictions of the binding site residues are verified by using several benchmark systems where the topology of the unbound protein and the bound protein-ligand complex are known. Predictions are made on the unbound protein. Agreement of results with the bound complexes indicates that the information for binding resides in the unbound protein. Cliques that consist of three or more residues that are far apart along the primary structure but are in contact in the folded structure are shown to be important determinants of the binding problem. Comparison with known structures shows that the predictive capability of the method is significant.

## Introduction

Ligand binding is generally known as a local process where the binding molecule finds a suitable location on the protein that has the right shape and the favorable energetic interaction [1]. However, observation of both short and long range conformational changes upon binding led to the suggestion that the full topology of the protein should be taking part in the ligand binding process [2]. According to this hypothesis, binding should depend not on the local structure, but rather on an interaction pathway on the protein that takes part in the collective reorganization of the residues to accommodate for the best and most favorable conformation of the protein-ligand complex. Numerous experimental observations are in support of this hypothesis. The changes in conformation in calcium binding proteins is cited in the first comprehensive review of this phenomenon [3]. All experimental evidence points out to the fact that the full topology of the protein should take part in such rearrangements. Thus, the information needed for determining the interaction pathway should somehow be hidden in the topology. In the simplest case, a coarse grained picture of the protein is satisfactory. The topology of the protein in this case is represented by the connectivity matrix, or the contact map, of the three dimensional structure, where the ij'th element of the matrix is unity if the ith and jth residues are in contact, and zero otherwise. Several successful models of proteins exist at this level of the topology, i e the residue based coarse grained topology. One of them is the Gaussian Network Model [4] which uses the connectivity matrix as its force constants matrix. In several recent papers [5], [6], [7], [8], using the GNM, we proposed a statistical thermodynamics argument that leads to the determination of the interaction path of the ligand binding problem. The method, which we term as the ‘maximum eigenvalue method’ [9] is based on determining the residues that exchange energy with their neighbors and the surrounding medium. In the present paper, we give several examples where we show that these residues which are closely associated with binding are located on paths of spatially contiguous residues. The concept of interaction pathways or networks in relation to ligand binding has been addressed from different perspectives. Lockless and Ranganathan [10] suggested that correlations between two residues resulting in energy transfer among them lead to interaction paths and are evolutionarily conserved. Nelson et al proposed a relation between long range perturbations and the interaction path [11]. Pan et al [12] and Amitai et al introduced the topological closeness measure as a determinant of interaction paths [13]. Our approach is an addition to this series of papers that emphasize the significance of topology in binding. The prediction of binding sites based on GNM is simple and easy to apply as demonstrated in the following examples, using thirty benchmark systems, presented in Table 1 and in the Supplementary data. A new additional concept that we introduce here is the ‘clique’, defined as a subset of three or more pairs of vertices, with each pair being connected by an edge, i.e. contacting (or interacting with) each other [14]. Cliques are expected to have great significance in protein-protein or protein-ligand interactions, as they are stiff regions, therefore likely to be conserved throughout evolution. In our data set, cliques made up of residue triads are identified since triads are frequently observed as spatial forms in the active sites of the proteins. We show the significance of cliques in relation to ligand binding.

**Table 1:** Six selected proteins from the test set.

FUNCTION	NAME OF THE PROTEIN	PDB CODE/CHAIN ID	
		Ligand-free state	Ligand-bound state
**Oxireductase**	Human Heme-Oxygenase-1	1NI6/B	1N3U/B
**Transferase**	Human glutathione transferase A1-1	1K3O/A	1K3Y/A
**Hydrolase**	Catalytic domain of Protein Tyrosine Phosphatase 1B	2HNP/A	1BZC/A
**Ligase**	BC Domain of Acetyl-coA Carboxylase2 (residues Val259–761Ala)	3GLK/A	3GID/A
**Lyase**	Human Carbonic Anhydrase II	2CBE/A	1A42/A
**Ca^+2^-binding Protein**	S100A6	1K9P/A	1K9K/A

## Results

### 1. Human Heme-Oxygenase-1

The first system that we analyze is an oxireductase, Heme oxygenase (HO) which is responsible for the degradation of heme to biliverdin. In the heme bound state, Human heme-oxygenase-1 (HO-1) arranges its helical shape with the help of highly conserved, distal helix residues, so that it supplies flexibility to accommodate substrate binding and product release [15]. Human HO-1 has a dynamic active-site pocket, which is enlarged in the apo state as distal and proximal helices surrounding the heme plane move farther apart. In the holo form, the active site residues Thr21, Val24, Thr23, Thr26, Ala28 and Glu29, which reside on the proximal helix, and Tyr-134, Thr-135, Leu-138, Gly-139, Ser-142, and Gly-143, which reside on the distal helix, are important as they interact with heme [16], [17]. According to the given crystal structure (PDB Code: 1N3U), the binding site for heme in the B chain contains the residues, Lys18, His25, Glu29, Gln38, Tyr134, Thr135, Gly139, Lys179, Phe207, Asn210 and Phe214. Phe207, Asn210 and Phe214 also lie on the proximal side of the active-site pocket. Below, we show that these specific features can be identified by applying the GNM to the apo form of the protein, i.e. 1NI6.pdb.

**Figure 1:**
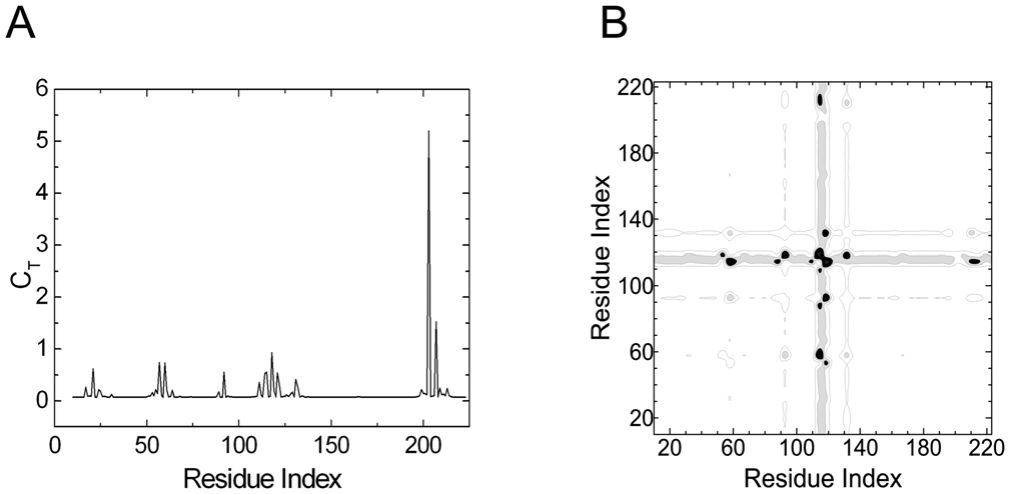
Important residues of human HO-1 predicted with GNM. a) Total correlation *C_T_* of residues as a function of residue indices. b) Contour plot of distance fluctuations 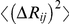 of 1NI6.pdb. Highest values indicated by black.

Figure 1a shows the total correlation, *C_T_*, of a given residue, presented as the residue index along the abscissa, obtained by using 1NI6.pdb. Figure 1b is the contour plot of the distance fluctuations where the residues that exchange energy with the surroundings are identified with a darker hue. The heavy vertical strip shows that the residues 118–124 interact with all the residues of the protein.

**Figure 2:**
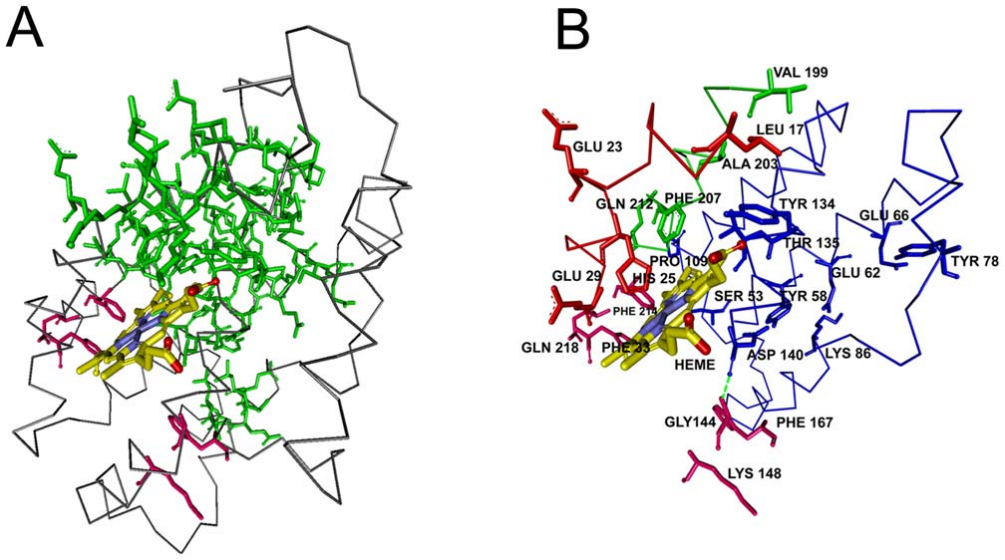
Three dimensional structure of one chain of human HO-1 chain. a) with Heme (yellow), interaction path (green) and the cliques (pink). b) Enlarged version showing interaction path residues and cliques (pink) with their labels. Green dashed line represents the hydrogen bond between Gly144 and Asp140.

In Figure 2a, the ligand and the residues on the interaction path, i.e. the set of residues with non-zero values of *C_T_*, are shown in yellow and green, respectively. Figure 2b is an enlarged version of figure 2a. Residues between 17 and 29 constituting the active site residues exhibit non-zero values of *C_T_*. The path that connects the surface to the heme starts with Leu17 and Glu23 at the surface and ends at His25 that neighbors the heme. The path is colored in red and the mentioned residues are labeled in Figure 2b. Residues 53–66 lie on helix H4 that contains the catalytic site Tyr58. The appearance of this region in Figure 1a is mostly due to its stability, resulting from hydrogen-bonded and electrostatic pair interactions with neighboring helix and loop structures such as Tyr58-Asp140, Glu62-Arg86, and Glu66-Tyr78 [18], [19]. Similar to the Leu17-His25 path, the residues between Pro109 and Thr135 form a path, one end of which is at the surface of the protein and the other with Tyr134 and Thr135 terminates at the heme.

The path that is lined by residues Pro109-Asp140 is colored in blue in Figure 2b. Finally, the largest peak corresponding to Ala203, which we define as the hub residue, and the second largest peak corresponding to Phe207, seen in Figure 1a, identifies the two residues neighboring the heme. The group of residues between Val199 and Gln212 are represented as the green path, most of which neighbor the heme molecule. All of the residues observed in Figure 1a are obtained from the apo structure, indicating that the information for binding is already present in the unbound structure.

The residues with non-zero total correlation values and that are in contact with the ligand, are presented in Table 2. The interaction path residues that are identified in Figure 2 are also presented in Table 3. Tabulating all the residues that lie on the pathways would lead to excessive detail. Therefore only residue pairs on the pathway separated by less than 7.2 Å are shown. Because of this, some of the residues cited in the text may not appear in Table 3, which we are presenting to supplement the information given here. The hub residues are identified in Table 3 with yellow highlight. As will be seen from Figure 2 and the following ones, the interaction paths do not consist of a single well defined line of contiguous residues, but rather of several bifurcating paths. Therefore, it is not possible to uniquely identify two extremities to an interaction path. The residues at the multiple extremities of the paths are defined as the gate residues.

**Table 2:** List of contacting residues.

1N3U/B	1K3Y/A	1BZC/A	3GID/A	1A42/A	1K9K/A
PHE207ASN210 PHE214	TYR9 ARG15ARG45GLN54 VAL55 GLN67 THR68 ARG69	CYS215SER216ALA217GLY218ILE219 GLY220ARG221GLN262	GLU593 ILE649 ASN679PHE704	GLN92 HIS94 HIS96 GLU117 HIS119	THR28 GLU33 ASP61 ASN63 ASP65 GLU67

**Table 3:** List of residue pairs along the interaction paths and the distances between them.

1N3U/B	1K3Y/A	1BZC/A	3GID/A	1A42/A	1K9K/A
i j dist	align="left" valign="top" tb="Single Width" tbw="10" tbc="#000000" bb="Single Width" bbw="10" bbc="#000000">i j dist	align="left" valign="top" tb="Single Width" tbw="10" tbc="#000000" bb="Single Width" bbw="10" bbc="#000000">i j dist	align="left" valign="top" tb="Single Width" tbw="10" tbc="#000000" bb="Single Width" bbw="10" bbc="#000000">i j dist	align="left" valign="top" tb="Single Width" tbw="10" tbc="#000000" bb="Single Width" bbw="10" bbc="#000000">i j dist	align="left" valign="top" tb="Single Width" tbw="10" tbc="#000000" bb="Single Width" bbw="10" bbc="#000000">i j dist
tb="Single Width" tbw="10" tbc="#000000">17 200 5.7	tb="Single Width" tbw="10" tbc="#000000">6 58 5.6	tb="Single Width" tbw="10" tbc="#000000">70 82 5.4	tb="Single Width" tbw="10" tbc="#000000">501 519 5.6	tb="Single Width" tbw="10" tbc="#000000">95 116 6.8	tb="Single Width" tbw="10" tbc="#000000">28 67 6.0
17 **203** 7.0	6 59 5.0	70 83 5.3	501 520 4.4	95 117 5.5	28 68 5.2
21 **203** 7.0	6 60 6.9	70 84 6.6	501 521 6.1	95 118 4.5	29 67 5.5
24 207 6.0	7 57 6.3	81 211 5.1	519 532 6.6	96 116 5.5	29 68 5.4
25 207 6.1	7 58 4.7	81 212 6.2	519 533 5.3	96 117 5.3	**31** 60 5.3
31 211 6.6	7 59 6.6	81 213 7.0	519 534 4.6	96 118 6.7	**31** 63 6.4
31 214 6.3	8 34 6.1	82 211 5.9	519 535 5.3	96 245 5.5	**31** 64 4.6
53 111 5.9	8 55 6.9	82 212 4.6	520 533 5.2	98 115 6.3	**31** 67 6.2
55 89 5.6	8 56 6.0	82 213 6.0	520 534 6.5	98 116 6.7	**31** 68 6.6
56 111 7.1	8 57 5.4	83 212 5.9	520 535 7.1	102 114 6.3	35 60 6.3
56 115 6.8	8 58 6.2	83 213 4.9	531 649 6.8	102 115 5.8	
57 114 6.9	9 34 5.5	83 **214** 5.8	531 650 5.6	103 114 5.8	
57 115 5.7	9 55 6.2	83 219 7.0	531 651 4.6	103 115 5.2	
60 115 5.9	9 **56** 5.6	83 222 6.8	532 649 6.0	104 114 6.1	
60 118 6.2	15 **56** 6.9	84 212 7.0	532 650 5.1	104 115 4.8	
60 119 6.4	15 68 7.1	84 213 6.2	532 651 6.5	104 116 4.6	
61 118 6.0	16 56 6.2	84 **214** 5.0	533 648 5.6	104 117 7.0	
64 119 6.3	19 72 6.4	84 217 6.6	533 649 4.7	104 245 6.1	
64 122 4.9	24 193 6.5	85 **214** 5.6	533 650 6.1	**105** 115 6.0	
111 213 6.6	24 194 6.8	85 215 6.3	534 595 7.0	**105** 116 4.5	
114 209 5.8	25 193 5.3	85 216 5.3	534 647 5.9	**105** 117 4.7	
128 199 5.0	25 194 5.2	85 217 4.1	534 648 5.3	**105** 147 6.8	
128 202 6.4	50 66 6.9	86 **214** 5.9	534 649 6.8	**105** 245 6.5	
131 199 5.9	55 66 6.8	86 215 5.8	535 647 6.5	114 147 6.2	
131 200 7.0	**56** 66 6.0	86 216 4.9	595 647 6.4	114 148 6.2	
131 202 5.9	**56** 67 6.9	86 217 6.4	647 706 5.4	114 149 5.7	
131 **203** 4.8	**56** 68 7.0	104 211 5.5	647 707 5.2	115 148 5.8	
132 **203** 6.4		104 212 6.8	647 709 5.3	115 149 5.3	
132 206 6.2		106 211 5.3	647 710 5.5	116 147 6.0	
		106 212 6.6	647 **713** 6.1	116 148 5.0	
		107 211 5.9	648 704 6.5	116 149 7.0	
		107 212 4.9	648 705 5.3	117 145 6.5	
		107 213 5.9	648 706 4.4	117 147 4.6	
		108 175 6.0	648 707 6.5	117 148 5.8	
		108 212 6.2	648 **713** 6.0	118 145 5.6	
		108 213 4.5	649 704 5.7	118 147 6.4	
		108 **214** 6.1	649 705 5.2	148 217 5.6	
		109 175 4.7	649 706 6.6	148 218 6.5	
		109 213 6.2	649 **713** 6.2	149 217 5.0	
		109 **214** 5.2	649 714 6.7	149 218 6.3	
		109 215 5.7	650 703 5.7		
		110 175 6.1	650 704 4.4		
		110 **214** 6.2	650 705 6.4		
		110 215 4.9	651 703 5.4		
		110 222 6.7	651 704 6.5		
		219 261 6.1	680 703 6.9		
		220 261 4.1	680 704 5.6		
		224 261 6.0	680 705 4.3		
			680 706 6.3		
			680 716 6.9		
			680 717 6.8		
			680 720 6.8		
			705 716 6.3		
			705 717 5.4		

**Residues shown in bold are the hub residues.**

Cliques of size three are shown in pink in Figure 2b. These are obtained at cut-off 6.2 Å, as 33Phe-214-Phe-218Gln and Gly144-Lys148-Phe167. The first triad is located on the proximal side while the latter lies on the distal side of the Heme molecule referring to the proximal and distal helices that sandwiches the Heme molecule upon binding[15]. Phe214 is a binding site residue while Gly144 is a highly conserved, catalytic residue [20]. Cliques obtained by a cut-off distance of 6.2 Å account for more than 50 percent of the highest conserved cliques of the proteins studied. In Table 4, residues with highest conservation for the six proteins that we present here are shown. These are obtained from the residue conservation data in PDBsum [21]. Among these, the highlighted residues are those belong to cliques of size three obtained by the 6.2 Å cut-off.

**Table 4:** Residues with high conservation.

**1NI6**: **129,130,131,132,133**,134,135,136,137,138,139
**1K3O**: 2,13,**23**,56,67,68,70,71,140,150,**154,156,157**
**2HNP**: 40,43–45,51,**56,57,**59,**66–70,82,83,85,87,91,94–96,98,**107,109,124,126,179,185,**213–218,220–223**,250,254,**257,262,266**
**3GLK**: 267–270,**274**,298,**300–303,305–307**,311,315,321,328,329,352,355,**356,361,** **373,374**,384,448,450,454–456,458,490,492,500,501,504,508,**517,518,521**,524, 528,529,**535**,562,565,567,580,582–584**,586–590,592–594,601,604**,675,683,**700–702,704,716**
**2CBE**: 5,**16,28–30,61,63,96,98,105–107,117,119,121,122,**186,**194,196–201,203, 205, 207,** 209, 222, **244**, 246,249,254,**259**
**1K9P**: 16,20,**29,33,61,65**,72

**Clique residues obtained by cutoff 6.2 Å are shown in bold.**

### 2. Human glutathione S-transferase

Glutathione S-transferases (GSTs) are involved in the catalysis of xenobiotics, carcinogens and conjugations with endogenous ligands. In addition, they can perform a variety of functions in metabolic pathways, which are not related with detoxification, such as the intracellular storage or transport of a variety of other hydrophobic, non-substrate compounds including hormones, metabolites, and drugs. Besides, due to the elevation of GST levels in tumor cells, they have been the focus of significant interest with regard to drug resistance [22], [23], [24], [25], [26]. In three-dimensional structures, a tyrosine or a serine has been shown to be central in catalysis [22], [23], [26], [27]. In addition, the side chain of Arg15 is thought to be involved in the inner coordination sphere of the sulfur[27].

**Figure 3:**
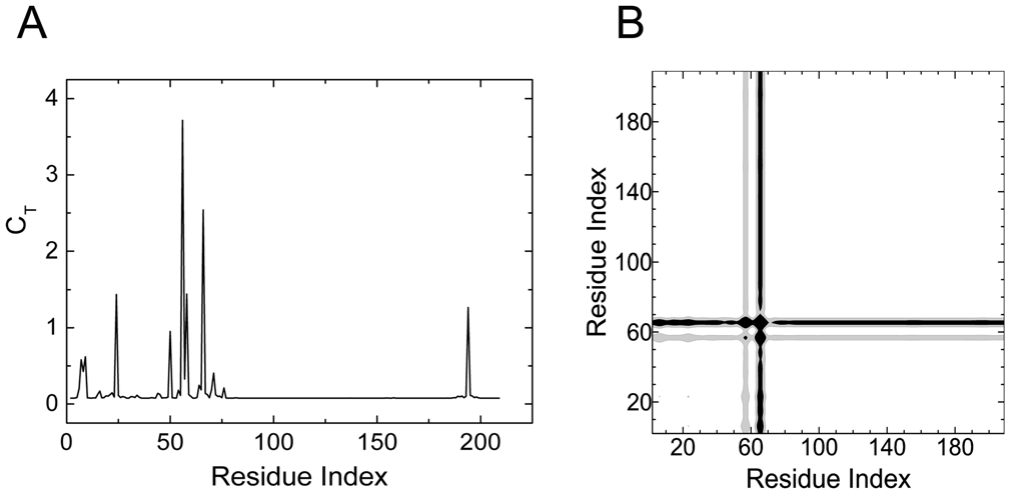
Important residues of human GST A1-1 predicted with GNM. a) Total correlation *C_T_* of residues as a function of residue indices. b) Contour plot of distance fluctuations 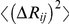 of 1K3O.pdb. Highest values indicated by black.

Figure 3a shows the total correlation, *C_T_* of residues. Figure 3b is the corresponding distance fluctuation correlation contour plot. The chain A of unbound crystal structure, 1K3O.pdb was used for calculations. Both chains are identical in sequence and in three dimensional structures, so are the ligands they bind. According to the given ligand-bound crystal structure 1K3Y.pdb, the binding site residues for S-hexyl-glutathione (GSH), are Tyr9-Arg45-Gln54-Val55-Pro56-Gln67-Thr68-Val111-Met208-Leu213-Phe220-Phe222 (chain A) and Ap101-Arg131 (chain B). In this structure, GST binds two glycerol molecules, as well.

**Figure 4:**
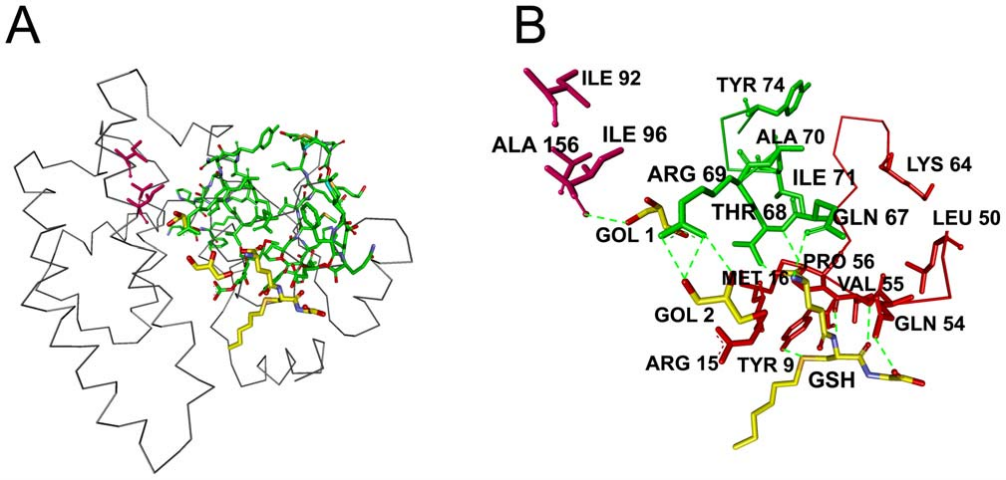
Three dimensional structure of one chain of human GST A1-1 chain A. a) with S-benzyl-glutathione (yellow), interaction path(green) and cliques (pink). b) Enlarged version showing interaction path residues and cliques (pink) with their labels. Dashed lines are the hydrogen bonds.

In Figure 4a, GSH and the residues on the interaction paths are shown in yellow and green, respectively. Figure 4b shows all of identified residues in detail. In Figure 4b, red colored residues line a path starting with a surface residue, Lys64 and ending with the binding site residues Tyr9 and Pro56, which is the hub residue. Tyr9 is conserved among the majority of known GSTs and it is emphasized as an important catalytic residue in literature [22], [23], [27], [28]. The three-dimensional structures have shown that the hydroxyl group of Tyr9 stabilizes the thiolate of GSH through hydrogen bonding [27]. Similarly, residues Leu50-Pro56 (also shown in red) form a shorter path, which has one end at the surface and the other end at the binding site. Three highly conserved residues Gln54, Val55 and Pro56, which also interact with the ligand via hydrogen bonds, play significant roles in the stability and function of the protein [29], [30]. Arg15 and Met16 are also interacting with the residues on the Tyr9 path. Arg15 is mentioned as an important active site residue in literature as well[31]. The residues between Gln67 and Tyr74 form another path, which is represented as the green path in Figure 4b, begins at the surface and ends where Gln67 and Thr68 are positioned to participate in hydrogen bonds with the amino group and γ-glutamyl carboxyl group of glutathione, respectively [32]. It involves five conserved residues Gln67, Thr68, Ala70, Ile71 and Tyr74[33]. A member of this path, Arg69 makes three hydrogen bonds with the second glycerol molecule.

The remaining binding site residues are situated on helix 9, which is known to be highly dynamic. Since, the region is assumed to become structured and localized upon ligand binding [28], [34], [35], its electron density is unresolved for apo human GST A1-1[36], [37]. Therefore, the binding site residues between Glu210-Phe222 do not appear in Figure 3a.

The residues Ala24 and Val194 display relatively high total correlations. They belong to two different secondary structures and are in contact with each other. Yet, in literature there is no comment on their contribution to the structure and function of the protein. These two residues are not shown in Figure 4b.

Cliques of size three, at cut-off 6.2 Å, are found as Ile92, Ile96 and Ala156 which are located near the interface of chain A and chain B. These three residues are shown in pink in Figure 4b. Unlike Ile92 and Ile96, Ala156 is a highly-conserved residue[33]. Ile96 is at the glycerol binding site and hydrogen-bonded to the first glycerol. (Figure 4b).

### 3. Tyrosine phosphotase

The protein tyrosine phosphatases (PTPs) work complementarily with protein tyrosine kinases in regulating signal transduction pathways which control many physiological processes, such as cell growth or cell differentiation[38], [39]. Protein tyrosine phosphatases display a great diversity both in structure and mechanism and they are recognized by the motif HCX5R at their active sites, with an essential cysteine residue (Cys 215 in PTP1B)[40], [41].

**Figure 5:**
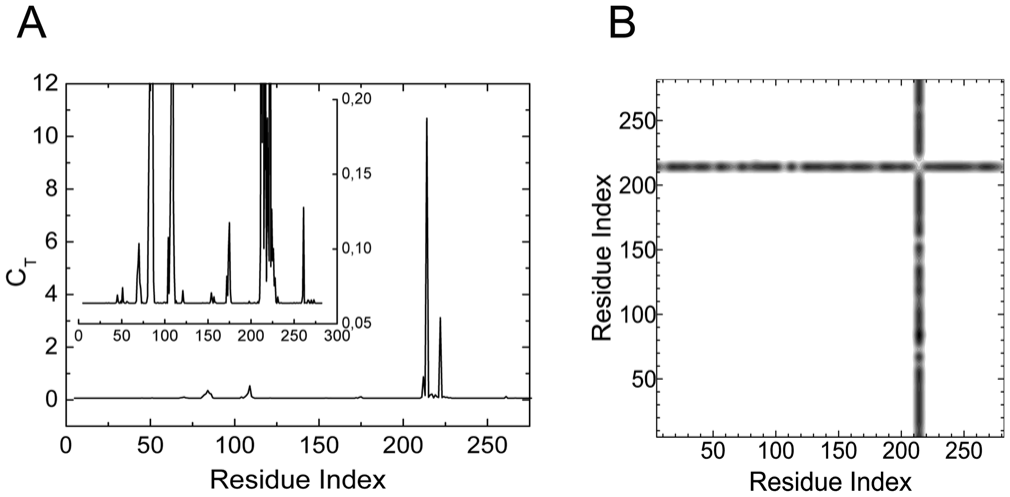
Important residues of human PTP 1B predicted with GNM. a) Total correlation *C_T_* of residues as a function of residue indices. b) Contour plot of distance fluctuations 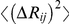 of 2HNP.pdb. Highest values indicated by black.

**Figure 6:**
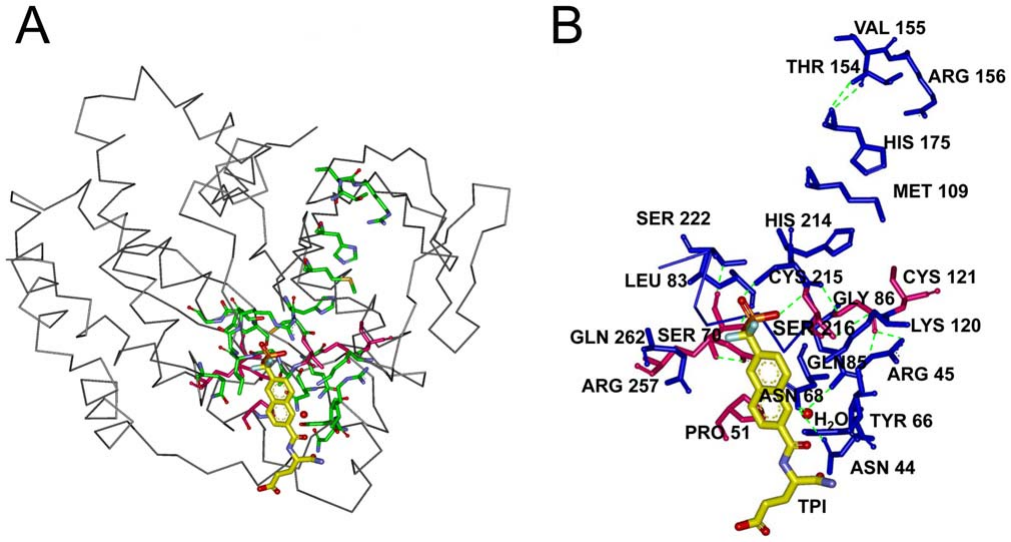
Three dimensional structure of one chain of human PTP 1B. a) with TPI (yellow), interaction path(green) and cliques (pink). b) Enlarged version showing interaction path residues and cliques (pink) with their labels. Dashed lines are the hydrogen bonds.

As seen from Figure 5a, the residues in between His214-Ser222 exhibit the highest total correlation, *C_T_*, where His214 is the hub residue. This group of residues is also observed in Figure 5b, the distance fluctuation matrix contour plot, to form a dark strip, implying that they are correlated with rest of the residues. The catalytic domain of PTP1B is composed of a single α/β domain, structured around a highly twisted β-sheet which spans the entire molecule. A-well known catalytic residue Cys215 is located on the loop that stays at the edge of this β-sheet. The His214-Ser222 region, which appear in total correlation plot (Figure 5a), indeed corresponds to the catalytic region of the protein [42]. In PTP1B, the residues His214, Cys215 and Ser 216 have central roles in the activation of the active-site [43]. Cys215 is emphasized as an important catalytic residue in literature [40], [41], [43]. In the inset of Figure 5a, the small peaks around the residues Arg45, Pro51, Tyr66-Asn68, Leu83-Gln85, Met109, Lys120, Thr154-Arg156, His175 and Gln262 can be seen. In addition to His214-Ser222 region, these mentioned residues draw an interaction path, which is shown in green in Figure 6a, around the ligand, which is shown in yellow in the same figure. In Figure 6b, the ligand and the interaction path is depicted in more detail and all residues with non-zero total correlation, *C_T_*, are labeled. All these residues are mentioned in literature. To start with, Arg45 sits in the loop where phospho-Tyr recognition occurs, with Pro51, a clique residue (shown in pink in Figure 6b). Being located in the binding site of PTP1B, Arg45 is also responsible from the electrostatic attraction of the ligand. Asn68 makes a hydrogen bond with Asn44 and it is located near a highly conserved residue, Arg257. Leu83 packs or surrounds the PTP loop (residues 213-223) where Gln85 makes a hydrogen bond with a highly buried water molecule. Residues Ile82-Pro87 (not shown in Figure 6b) form the core structure that surround the PTP loop. Residues around Met109 form the hydrophobic core structure and they are less conserved compared to the Ile82-Pro87 motif. Lys 120 is another binding site residue, which H-bonds to Ser216 and interacts with Asp181 (not shown in Figure 6b), known as a general acid catalyst among the vertebrate PTPs. Arg156 is conserved more than %80 among all vertebrate PTP domains. His175 is found in the surface exposed WPD loop (residues His 175–Val 184), where a major conformational change takes place upon binding of phosphopeptides to the PTP loop. The PTP loop then, moves several angstroms to close the active site pocket and trap the bound phosphotyrosine [44]. The WPD loop is also not shown in Figure 6b. Gln262 is also actively involved in ligand-binding process [43].

Cliques of size three are found as Pro51-Ser70-Arg257 and Gly86-Cys121-Ser216 at cut-off 6.1 Å, all of which are highly conserved (pink residues in Figure 6b). The first triad is located around the active site; Pro51 is on the phosho-Tyr recognition loop and Arg257 is on the loop Leu250-Leu267 that spans the active site [45]. Arg257 makes a hydrogen bond with the PTP loop and also believed to be involved in stabilization of the nucleophilic nature of the active site cysteine, Cys215[36]. Cys121, another clique residue is interacting with Cys215, as well[36]. It has been previously reported that Cys121 in PTP1B is a highly nucleophilic group accessible and ready for covalent attachment of 1,2-NQ, which is a known inhibitor of PTP1B. It causes considerable reduction in dephosphorylation activity of PTP1B. Moreover, Cys121 was reported as a non-active site cysteine residue, but it sits on an allestoric site, where it can inhibit the enzyme activity through specific mechanisms [35], [45]. There are a number of PTPs in which Cys121 (90%) is highly conserved [44]. Ser216 lies on the active site and functions in the activation of Cys215[43].

All of the residues observed in Figure 5 and Figure 6 are obtained from the apo structure, 2HNP.pdb.

### 4. Biotin Carboxylase Domain of Acetyl-CoA Carboxylase 2

Acetyl-CoA Carboxylase (ACC) is responsible from the biotin-dependent synthesis of malonyl-CoA, through its catalytic domains, biotin carboxylase (residues Val259–761Ala) and carboxyltransferase (residues Leu1809–Gly2305). [46] Since it has a crucial role in fatty acid metabolism, ACC has become a target for therapeutic intervention against the treatment of diseases such as type II diabetes, cardiovascular diseases and atherosclerosis, metabolic syndrome in general, and in the control of obesity[47], [48], [49], [50]. Acetyl-CoA Carboxylase 2 (ACC2) in mammals is expressed in the heart and skeletal muscle cells where it regulates the fatty acid oxidation via its malonyl-CoA product [50], [51], [52], [53], [54]. Therefore, the inhibitors of ACC2 may be used as novel anti-obesity drugs or therapeutic agents against the metabolic syndrome[50], [51], [52], [53], [54], [55]. Among currently known small potent inhibitors of mammalian ACCs, only Soraphen A binds to an allosteric site which is about 25 Å distant from the active site of the biotin carboxylase (BC) domain[56], [57].Soraphen A interacts extensively with the BC domain where it is in contact with highly conserved residues [50].

**Figure 7:**
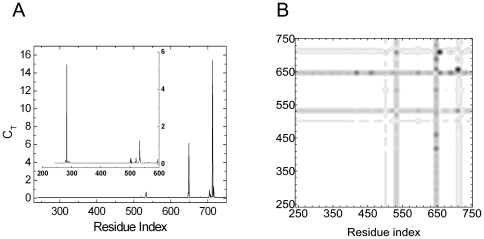
Important residues of BC domain of ACC2 predicted with GNM. a) Total correlation *C_T_* of residues as a function of residue indices. b) Contour plot of distance fluctuations 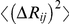 of 3GLK.pdb. Highest values indicated by black.

In its crystal structure, (PDB code: 3GID), where Sarophen A is bound to the human ACC 2, the binding site residues are given as Lys274-Ser278-Arg277-Glu593-Met594-Asn599-Asn679-Trp681-Phe704-Trp706. Figure 7a shows total correlation, *C_T_* as a function of residue index and the residues between Phe704–Ser715, exhibit non-zero values of *C_T_*, obtained by using 3GLK.pdb. Figure 7b is the contour plot of the distance fluctuations. Residues that exchange energy with the surroundings are shown in black. The intense vertical strips indicate that the residues around Gln504, Glu533, Ile649 and Ala713, which is the hub residue, are able to communicate with the rest of the protein.

**Figure 8:**
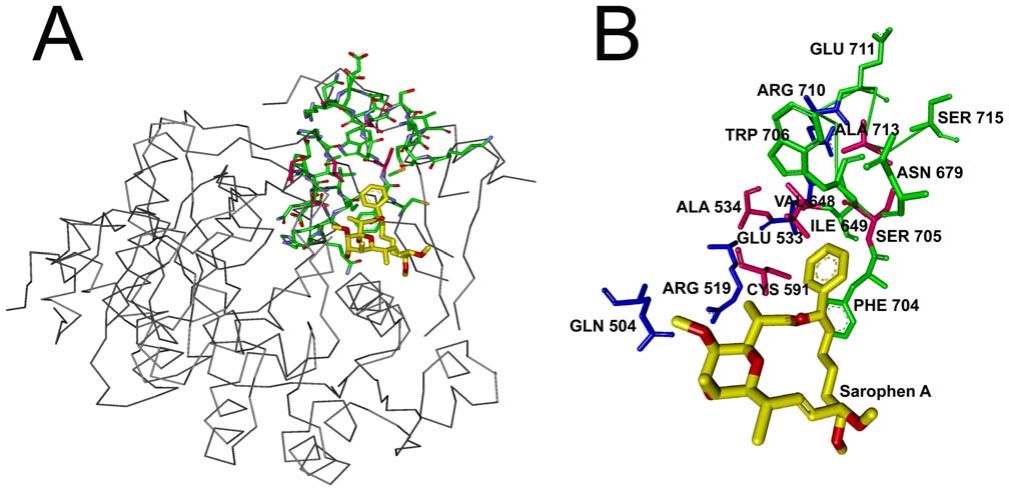
Three dimensional structure of BC domain of ACC2. a) with Soraphen A (yellow), interaction path (green) and cliques (pink). b) Enlarged version showing interaction path residues and cliques (pink) with their labels. Dashed lines are the hydrogen bonds.

Figure 8a shows the ligand, Soraphen A, in yellow and the interaction path in green. Figure 8b, is a more detailed version of Figure 8a where all identified residues are labeled. As it can be seen from Figure 8b, Glu711, a surface-exposed residue, sits where the green path starts. This green path terminates at Phe704 and Val648, which is also a clique residue (shown in pink in Figure 8b). Phe704, with Ser705 and Trp706, surrounds the ligand. Ile649 and Asn679 are located around the Phe704–Ser715 path. Ile649 appears with the second highest *C_T_* value, according to Figure 7a. The blue path (Figure 8b), which starts with Arg710, involves Glu533, Arg519 and ends with Gln504. Glu533 is a well-conserved residue[33]. There are small peaks around the 500^th^ and 520^th^ residues, which may correspond to the residues Gln504 and Arg519, lying in blue path. These two residues are known as catalytic residues in ACC2 [33], [58].

In this paper, we present no more than the fastest mode results for total coupling of residues. Yet, we checked the results for the second and the third fastest modes and identified new paths of same kind which extend from surface to the ligand binding (active site) pocket. For instance, residues around Ser278 show the highest total correlation values in the fastest third mode. Lys274, Ser278 and Arg277 indeed stabilize the ligand via hydrogen bond formation[33]. Results for the second mode are presented in the inset of Figure 7a. Yet, these residues are not shown in Figure 8b. We will present the contributions from higher modes in detail in our future work.

Cliques of size three, at cut-off 6.1 Å, are found as Ala534-Cys591-Val648 and Val648-Ser705-Ala713. Clique residues which are shown in pink in Figure 8b, reside either in close proximity or within the active site pocket, most of which fall on the interaction paths. All clique residues are highly conserved residues[33].

### 5. Human Carbonic Anhydrase II

Carbonic anhydrases are found almost in all organisms, and they are used as catalysts in reversible hydration of carbon dioxides. Zn^+2^ ions are essential for their catalytic activity which can bind four or more ligands in carbonic anhydrases. Three coordination sites are occupied by the imidazole rings of the His residues and the forth coordination site is occupied by a water molecule or a hydroxide ion [59]. Carbonic anhydrase II, which is a major element of red blood cells, is one of the most active carbonic anhydrases and has been the most widely studied. It has evolved as a proton shuttle with the primary component His 64 [59]. The catalysis of carbon dioxide hydration by carbonic anhydrase, so the reaction rate, depends heavily on pH. The enzyme is more active in high pH values [59].

In its crystal structure (PDB code: 1A42), human carbonic anhydrase II is complexed with the drug used for glaucoma therapy, the sulfonamide inhibitor brinzolamide. The given binding site residues are His64-Gln92-His94-His96-His119-Val121-Phe131-Val135-Leu198-Thr199-Thr200.

**Figure 9:**
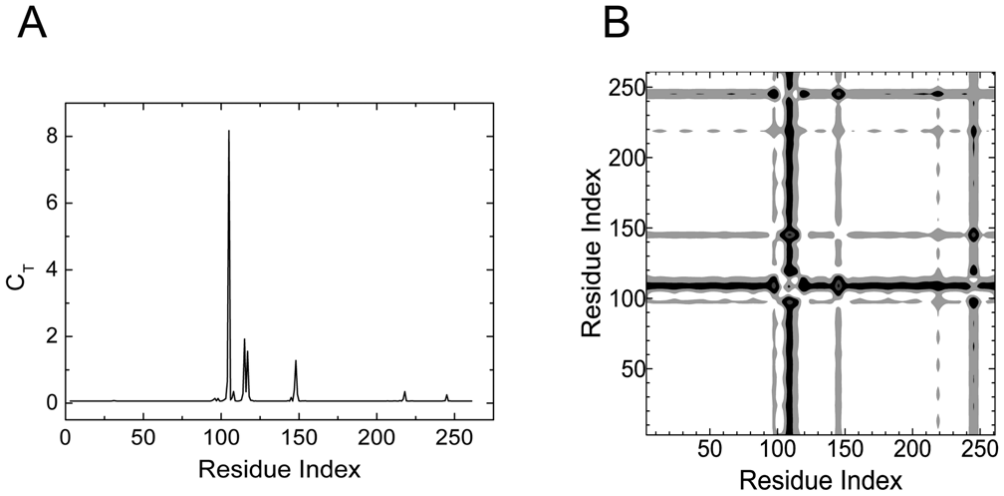
Important residues of Carbonic anhydrase II predicted with GNM. a) Total correlation *C_T_* of residues as a function of residue indices. b) Contour plot of distance fluctuations 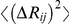 of 2CBE.pdb. Highest values indicated by black.

Residues between His96–His107, Tyr114–His119, Phe147–Lys149, Ser217–Val218 and Asn244–Arg246 exhibit non-zero values of total correlation according to Figure 9a. These residues interact with all residues of the protein, referring to the contour plot of the distance fluctuations given in Figure 9b. These two plots are obtained using the unbound structure of the protein. (PDB code: 2CBE).

**Figure 10:**
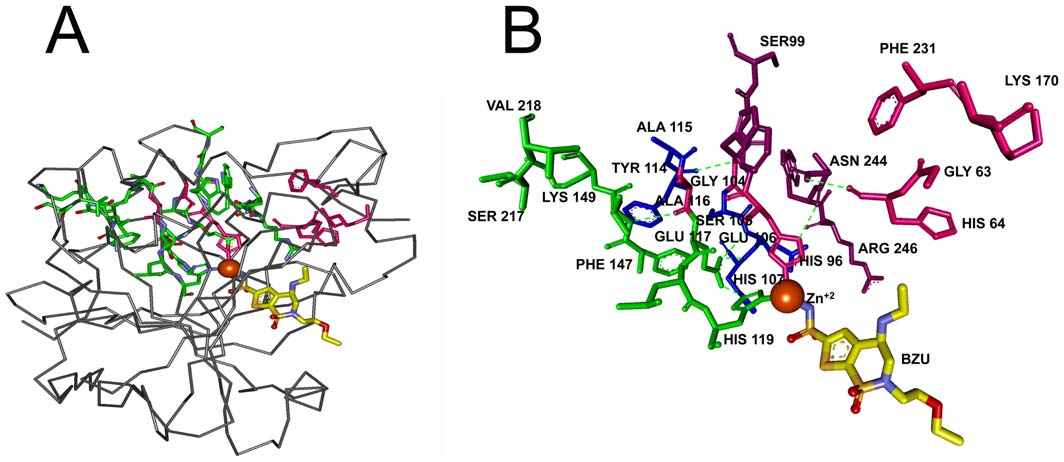
Three dimensional structure of Carbonic anhydrase II. a) with Brinzolamide (yellow) and Zn+2(orange), interaction path (green) and cliques (pink). b) Enlarged version showing interaction path residues and cliques (pink) with their labels. Dashed lines are the hydrogen bonds.

In Figure 10a, the ligand and the residues on the interaction paths are shown in yellow and green, respectively. Figure 10b depicts all of identified residues in detail. The first path, which is colored in green in Figure 10b, has one end at Ser217–Val218 and Lys149, and the other end at His119. The blue path starts with Ala115 and ends where the two paths are merged by the H-bonds Glu106 and His107 make with Glu117. Through the path Ala115 also interacts with Gly104 via hydrogen bonding. Ser105, which is the hub residue, links Gly104 with Glu106 and His107. The purple path has surface exposed Ser99 at one end and terminates at His96, which interacts with the Zn^+2^ ion that is directly bound to the ligand (Figure 10b). Indeed, the active site cleft is characterized by this Zn^+2^ ion which is tetrahedrally coordinated by N atoms of three histidine residues His94(not shown in Figure 10b), His96 and His119 and a water/hydroxide molecule [60]. Ser105 and Glu117 are within the 10 residues that are completely invariant among the whole family of α-CAs and α-CA-related proteins. Ser105 is involved in stabilizing the protein structure, while Glu117 function as an indirect ligand in the active enzyme [61]. Asn244 and Arg246 are two conserved residues, (colored in purple in Figure 10b) which also neighbor the ligand [33].

Cliques of size three are calculated at cut-off 6.1 Å. The residue triads His96-Gly104-Ala116 and Gly63-Lys170-Phe231 appear around the catalytic site of the protein (pink residues in Figure 10b). His96 is an important residue which interacts with the Zn^+2^ ion during the catalysis. Gly104 and Ala116 are located in a conserved region, which involves Ser105 and Glu117 [61]. Gly63 is next to His64 which acts as a protein shuttle during catalysis[59]. The side chain of Lys170, the closest of all other residues to the pathway for protein transfer with His64 in the outward orientation [62]. It is believed that one function of Lys170 is to maintain an environment of His64 that maximizes protein transfer and catalysis of the hydration of CO_2_ and dehydration of bicarbonate, by keeping it in its outward orientation [63]. In the outward conformation, the imidazole ring of His64 heads out of the active site cavity and the hydrophobic residue Phe231 is located near that cavity.

### 6. S100A6

S100 proteins are small dimeric proteins which belong to the EF-hand family of calcium-binding proteins. They are characterized by a pair of calcium-binding sites each having the helix-loop-helix structural motif. Upon calcium binding, the conformation of the protein changes through a hand-type motion, which renders the angle between the helices of EF2 from negative to positive [64].

The expression of S100 proteins is cell and tissue-specific. Most S100 genes are localized within human chromosome 1q21[65], a region which is susceptible to changes during tumor progression in transformed cells. [66] The expression of the S100A6 gene, is particularly increased in leukemia cells [67] and during the G1 phase of the cell cycle [68], which implies its role in cell cycle progression. Experiments at the protein level also show that S100A6 may be involved in cell growth, cell differentiation and motility [69], [70], [71], [72].

**Figure 11:**
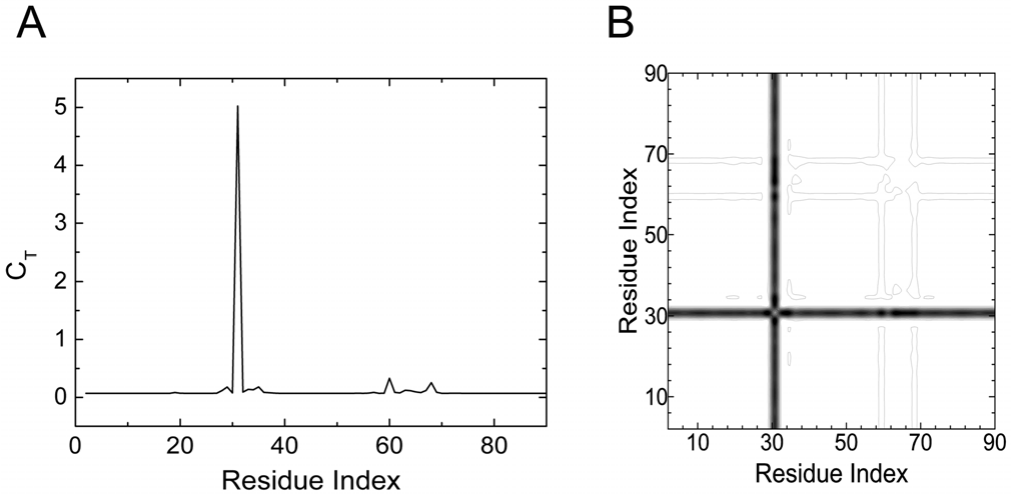
Important residues of s100A6 predicted with GNM. a) Total correlation *C_T_* of residues as a function of residue indices. b) Contour plot of distance fluctuations 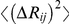 of 1K9P.pdb. Highest values indicated by black.

In the crystal structure of human S100A6 (PDB code: 1K9K), binding sites for Ca^+2^ ions are given as, Ser20-Glu23-Asp25-Thr28-Glu33 and Asp61-Asn63-Asp65-Glu67-Glu72. In Figure 11a, it is observed that residues between Thr28-Lys35, which contains the hub residue Lys31, and Asp61-Glu67 exhibit non-zero values of total correlation, *C_T_*. Figure 11b shows the contour plot of the distance fluctuations where the residues that exchange energy with the surroundings, are identified with a darker hue. The heavy vertical strip shows that especially the residues 28–33 interact with the rest protein.

**Figure 12:**
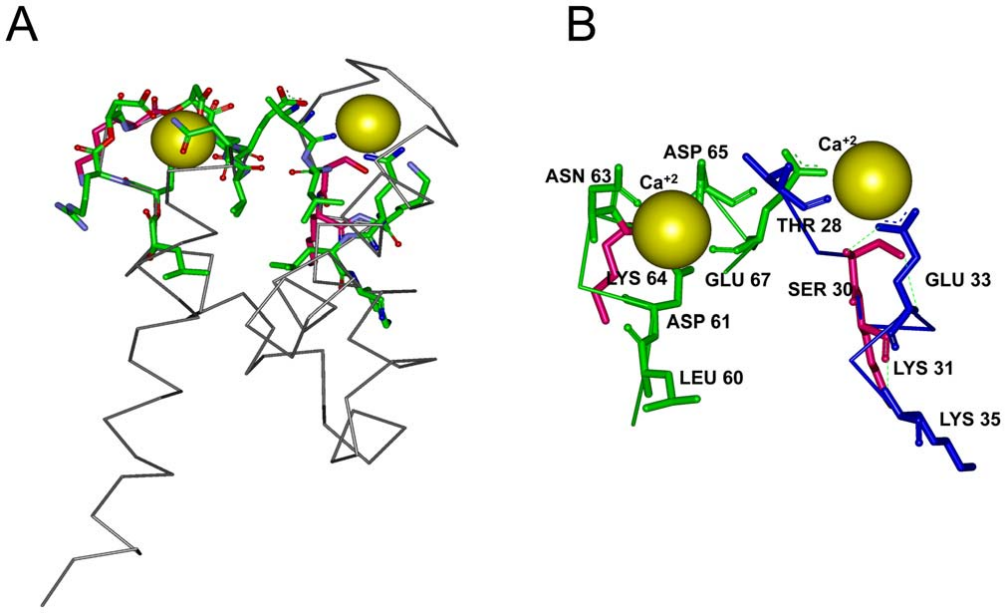
Three dimensional structure of one chain of S100A6. a) with bound Ca^+2^ ions (yellow), interaction path (green) and cliques (pink). b) Enlarged version showing interaction path residues and cliques (pink) with their labels. Dashed lines are the hydrogen bonds.

In Figure 12a, the ligand and the residues lining the interaction paths are shown in yellow and green, respectively. Figure 12b is an enlarged view of the ligand and the interaction paths through the protein. In human S100A6, secondary structure elements are arranged into two calcium binding motifs, which compromise Ca^+2^ binding site I and site II. For site II (S100-hand motif), the most noticeable difference, upon Ca^+2^ binding is the movement of Glu33. In contrast, the coordination of the Ca^+2^ in site I (EF-hand motif), is largely mediated by main chain carbonyl of Glu67 and the side chains of Asp61, Asn63, Asp65 and Glu72. [73] As shown in Figure 12b, residues between Thr28-Lys35 form a path beginning with hydrogen bonded residues Lys35 and Lys31, that terminates with two binding site residues Thr28 and Glu33. The path that surrounds site I is shorter and involves Asp61, Asn63 and Glu67, which indeed begins with Leu60, a well-conserved surface-exposed residue[33].

According to our results, obtained by using the unbound structure, 1K9P.pdb, the residue pairs with the highest total correlation appear around the residues Lys31 and Leu60. Interaction path residues are mostly the binding site residues. Other residues line a network through the protein between the two Ca^+2^ binding sites. (Figure 12a) Cliques are calculated at cut-off 6.1 Å and shown in pink in Figure 12b. The triad Lys 31-Leu 60-Lys 64 also appears around the catalytic site of the protein.

Results for the remaining twenty four systems are provided in the Supporting Information S1.

## Discussion

Based on the GNM, structural and thermodynamic features of the bound state are predicted by using the unbound structures. This shows that the binding information is already present in the unbound structure. This was also observed by us in a recent work [6].

We have presented a collection of computational techniques to study the relationship between the 3-dimensional structure and the dynamics of protein. These two methods relate protein structure with protein function and protein dynamics in terms of ligand binding. Contact map of a protein can be investigated by the tools of graph theory and provides information about the stiff and conserved, therefore functionally important regions. These certain regions are the cliques made up of residue triads and they typically reside either along the catalytic region, if the protein is an enzyme, or along the ligand binding pocket. This kind of approach establishes the structure-function correlations in proteins. Gaussian Network Model (GNM), on the other hand, correlates the fluctuations of residues with the three dimensional structure of the protein. The two computational methods are applied to the crystal structures of known systems. Ligand-free structures are used to find the cliques and the interaction pathways through which the energy is transferred to the system. Then, ligand-bound systems are used as positive controls.

We conducted our study in a diverse set composed of 30 proteins each having a distinct function. Among those we obtained successful results in 29 systems. Residues with non-zero total correlation (C_T_) values appear along a path with one end located at the surface and the other end exposed to the ligand binding pocket (site). These residue interaction networks indicate the existence of the interaction path which is directly related with ligand binding and highly dependent on the topology of the protein. In this paper, we present no more than the fastest mode results for total coupling of residues. Yet, we checked the results for the second fastest mode and identified new pathways of same kind which extend from different energy gate residues (to the ligand binding pocket).

In a limited set of six proteins, presented in Table 4, several cliques made up of residue triads, obtained by a cutoff distance of 6.2 Å, appear as conserved residues. For other proteins, presented in Supporting Information S1 we saw that cutoff distances around but not exactly equal to 6.2 Å were needed for favorable agreement of the predictions with experimental observation. Thus, a clear-cut specification of a clique-cutoff distance is not available, at least within the level of approximation of the present model. However, the shortcoming due to lack of a single cutoff value notwithstanding, we can say from the data we analyzed that several of the catalytic residues which are emphasized in the literature are predicted by the present Gaussian model.

Our approach exhibits a high predictive capability. Table 1 involves the data set and the summary of results for the remaining proteins is presented in Supporting Information S1. We have shown this approach to be successful in the identification of interaction pathways and conserved regions in a diverse set of protein-ligand systems.

## Methods

A coarse grained GNM analysis based on 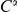 atoms of residues and a harmonic potential is used. The position of the ith 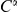 is denoted by 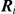. The 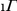 matrix of GNM is defined as
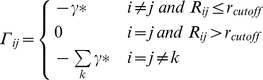
(1)


Here, 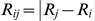 is the distance between residue i and j, r_cutoff_ is the distance that defines the neighborhood condition generally taken between 6.5–7.5 Å. 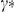 is a positive scaling parameter. The correlation of fluctuations follows from the harmonic assumption as 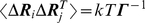
(2)where, 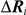 is the fluctuation vector of the ith 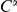, 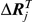 is the transpose of the fluctuation vector of the jth 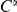, *k* is the Boltzmann constant and *T* is the temperature. The correlation matrix may be expressed in modal form as [74]

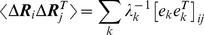
(3)where, 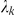 is the kth eigenvalue of the 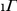 matrix, 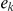 is the corresponding eigenvector, and 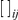 is the ijth element of the enclosed matrix. In our recent work [8] we considered only the largest eigenvalue component of the 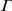 matrix for a comparative study of various HLA proteins.

The mean square fluctuations of the distance between residue i and j is then written as
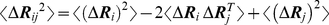
(4)


The correlation of residue fluctuations with an energy exchange 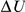 of the protein is 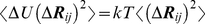
(5)


Equation 5 now shows the correlations of energy fluctuations with the squared fluctuations of the distance between residues i and j [8]. Summing both sides of Eq. 5 over the jth index leads to the total coupling 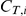 of residue i to its surroundings
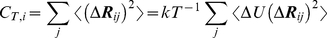
(6)


The last term in Eq. 6 acknowledges the role of energy exchange of residue i with its surroundings that consist of the neighboring residues and the surroundings of the protein. Our exploratory calculations showed that there is a small dependence on the cutoff value, usually taken as 7 Å as the radius of the first coordination shell for C_α_ atoms. In the present study, in order to eliminate, or at least minimize, this dependence, we averaged the 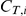 values over the interval 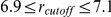 measured in Angstorms. The lower and upper values are selected by trial and error. If 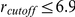, then some relevant interactions are not taken into account. If, on the other hand, 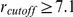, then too many residues all of which do not lie on the same path result that are not of interest to the binding problem are included.

In the largest eigenvalue formalism, the set of residues with non-zero values of 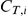 constitute the interaction pathway. As has been shown before [8], and as will also be shown below, these residues are in contact with each other, in general, and constitute a path, the ends of which are exposed to the surroundings of the protein, which we termed as energy gates. Along this path lies a residue that is highly interactive with a large number of residues of the protein, and hence is referred to as the hub.

By its structural nature, a clique constitutes a stiff region of the protein. Considering the contact matrix ***A*** of the protein, cliques of size three are obtained according to the following recipe
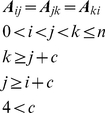
(7)where i, j and k are residue indices, c is the residue distance (number of residues) between contacting residues, and n is number of residues for each protein.

We studied several different systems, six of which we selected in the present study. These are given in Table 1. The last two columns of Table 1 give the pdb codes of the ligand free and ligand bound structures. In all our calculations, we perform the predictions on the ligand-free structure and compared the results using the ligand-bound structure.

The cutoff distances for the 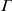 matrix were chosen as follows: Using 4810 non-redundant PDB structures obtained from Reference [75], we counted the frequency of observation of residue contacts for different values of 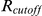, which was varied in the interval 5–15 Å. The results are shown in the first figure of Supporting Information S2, where the filled circles are the results of calculations. The straight line drawn to the linear part of the curve therein shows the scaling region. In this region, changing the 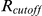 value by a factor changes the number of observations proportionately, and this relates simply to the size effect. Below the scaling region, effects other than size effects are accounted for as 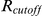 is increased. An 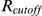 at the boundary of the non-scaling and scaling regions reflects all the effects that are of interest. The arrow in the first figure of Supporting Information S2 corresponds to an 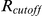
value of 7.2 Å. In order to include effects that would come from smaller 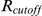 values, we took five equally spaced stations between 6.9–7.2 Å, and averaged the reported total correlation values over these five stations.

The cutoff distances for the cliques were chosen with a similar analysis described in the preceding paragraph for the contacting residue pair's analysis. The results are shown in the second figure of Supporting Information S2, where the filled circles are the results of calculations. The straight line drawn to the linear part of the curve shows the scaling region. An 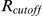 has to be chosen below the scaling region. The arrow in the figure corresponds to an 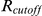 value of 6.2 Å. In the calculations, we tried 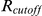 values of 6.0, 6.1, 6.2, 6.3 and 6.4 Å, and accepted the value of 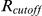 that led to the most consistent comparison of the model with observations.

The binding site residues using the bound complexes are defined as follows: In the complex, if the distance between an atom of a residue and an atom of the ligand were less than 3.5 Å, and if this residue had a non-zero total correlation calculated by using the unbound PDB file, then the residue was defined as a contacting residue. The list of contacting residues for the six systems analyzed in this study is given in Table 2.

## Supporting Information

Supporting Information S1 Total correlation *C_T_* of residues as a function of residue indices and the corresponding three dimensional structures showing the nteraction paths and the cliques for the 24 benchmark proteins.
(DOC)

Supporting Information S2Log-Log plots of the relation between Number of residue-residue contact versus 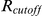
and of the relation between number, N, of cliques versus 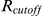.(DOCX)
