# Supplementary material for: Identification of Ligand Binding Sites of Proteins Using the Gaussian Network Model
Source: PLoS One. 2011 Jan 25;6(1):e16474. doi: 10.1371/journal.pone.0016474 (PMC3026835; doi:10.1371/journal.pone.0016474)
Supplement: Supporting Information S1 — Total correlation CT of residues as a function of residue indices and the corresponding three dimensional structures showing the nteraction paths and the cliques for the 24 benchmark proteins. (DOC) [file pone.0016474.s001.doc]

Table1 – Summary of results for the whole data set

| PROTEIN | Clique cutoff(Å) | PDB Code/Chain ID | | Success in Predicting Binding Site |  | |
| --- | --- | --- | --- | --- | --- | --- |
|  |  | Ligand-free state | Ligand-bound state |  |  | |
| Oxireductases | | | | |  | |
| Alcohol Dehydrogenase | 6.0 | 1E3E/A | 1E3I/A | Yes |  | |
| Heme oxygenase- 1 | 6.2 | 1NI6/B | 1N3U/B | Yes |  | |
| Ispc | 6.0 | 1ONN/A | 1ONP/A | Yes |  | |
| Transferases | | | | |  | |
| Adenosine Kinase | 6.0 | 2PKF/A | 2PKK/A | Yes |  | |
| Glutathione S-transferase | 6.2 | 1K3O/A | 1K3Y/A | Yes |  | |
| Map Kinase P38-α | 6.1 | 1WFC/A | 1OVE/A | No |  | |
| Kinase Domain TRP-Ca Channel | 6.1 | 1IAJ/A | 1IAH/A | Yes |  | |
| Cyclin-Dependent Kinase | 6.2 | 1HCL/A | 1HCK/A | Yes |  | |
| Hydrolases | | | |  |  | |
| M-phase inducer phosphatase 2 (Cdc25b) | 6.1 | 1CWR/A | 1CWS/A | Yes | | |
| Angiogenin | 6.0 | 1ANG/A | 1GV7/A | Yes | | |
| Carboxypeptidase A | 6.1 | 5CPA/A | 7CPA/A | Yes | | |
| Gamma Chymotyrpsin | 6.0 | 2GCH/B and C | 1AB9/B and C | Yes | | |
| Glyoxalase I | 6.1 | 1FA8/A | 1FA5 | Yes | | |
| Lysozyme | 6.1 | 1REX/A | 1REY/A | Yes | | |
| Tyrosyl-DNA phosphodiesterase | 6.1 | 1JY1/A | 1MU7/1 | Yes | | |
| Beta-lactam synthetase | 6.1 | 1M1Z/A | 1MB9/A | Yes | | |
| Protein Tyrosine Phosphatase (PTP1B) | 6.1 | 2HNP/A | 1BZC/A | Yes | | |
| Vacuolar protein sorting Protein29 (VPS29) | 6.1 | 1Z2X/A | 1Z2W/A | Yes | | |
| Phospholipase C | 6.4 | 1PTD/A | 1PTG/A | Yes | | |
| Pancreatic α-amylase | 6.0 | 2QMK/A | 2QV4/A | Yes | | |
| Ligases | | | | | | |
| Acetyl-CoA carboxylase (ACC) | 6.1 | 3GLK/A | 3GID/A | Yes | |  |
| Lyases | | | | | |  |
| Carbonic Anhydrase II | 6.1 | 2CBE/A | 1A42/A | Yes | |  |
| Ferrochelatase | 6.4 | 1AK1/A | 1C1H/A | Yes | |  |
| Hydroxynitrile Lyase | 6.1 | 1DWO/A | 1DWP/A | Yes | |  |
| Non-enzymes | | | | | |  |
| Adipocyte Lipid binding Protein | 6.8 | 1G7N/A | 1G74/A | Yes | |  |
| Ca-Binding S100A6 | 6.1 | 1K9P/A | 1K9K/A | Yes | |  |
| Copper Resistance Protein | No clique! | 3DSP/A | 3DSO/A | Yes | |  |
| L-leucine Binding Protein | 6.0 | 1USG/A | 1USK/A | Yes | |  |
| Fibrillin | 6.2 | 1UZQ/A | 1UZJ/A | Yes | |  |
| TNF receptor associated factor (Traf 6) | 6.5 | 1LB4/A | 1LB5/A | Yes | |  |

**TOTAL CORRELATION GRAPHS and FIGURES for the remaining PROTEINS in DATA SET**

1. **Oxireductases**

**
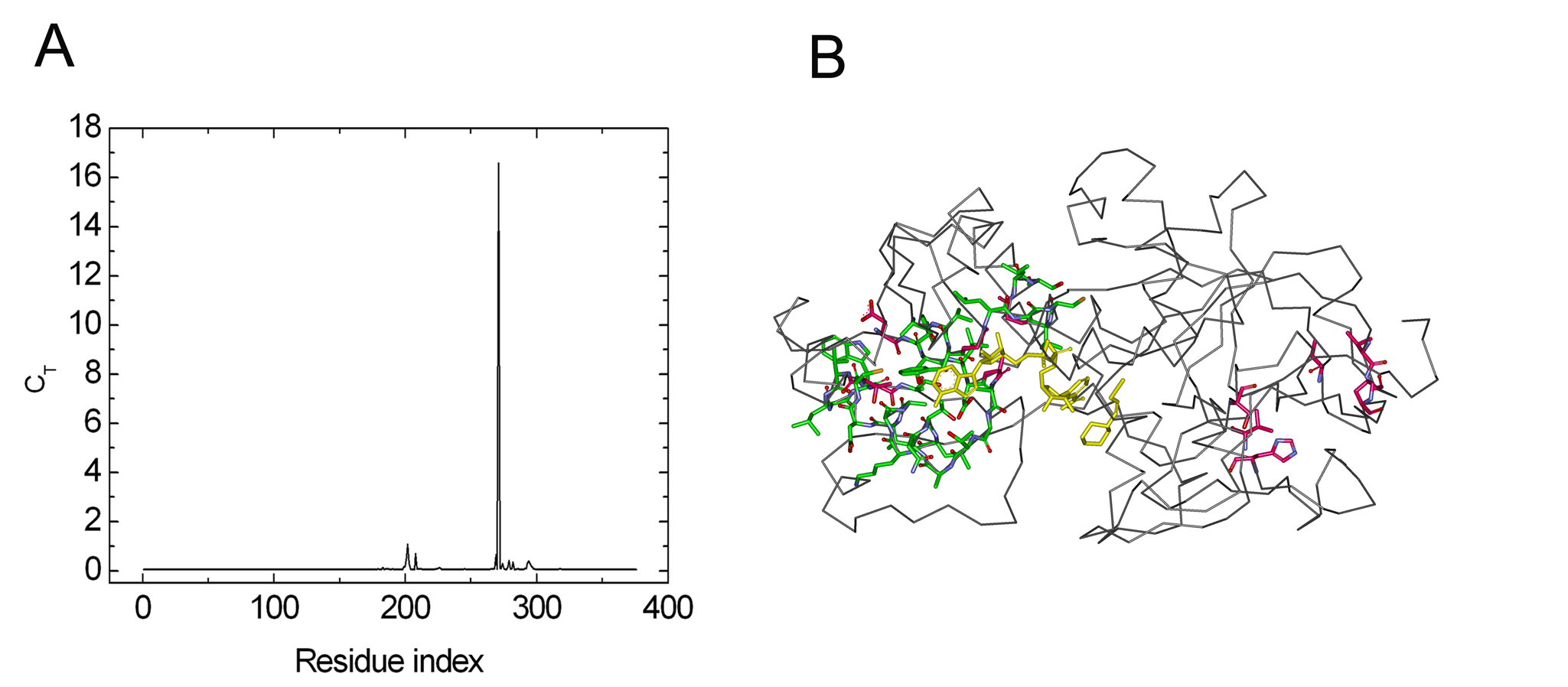
**

**Figure 1 Alcohol Dehydrogenase**

**
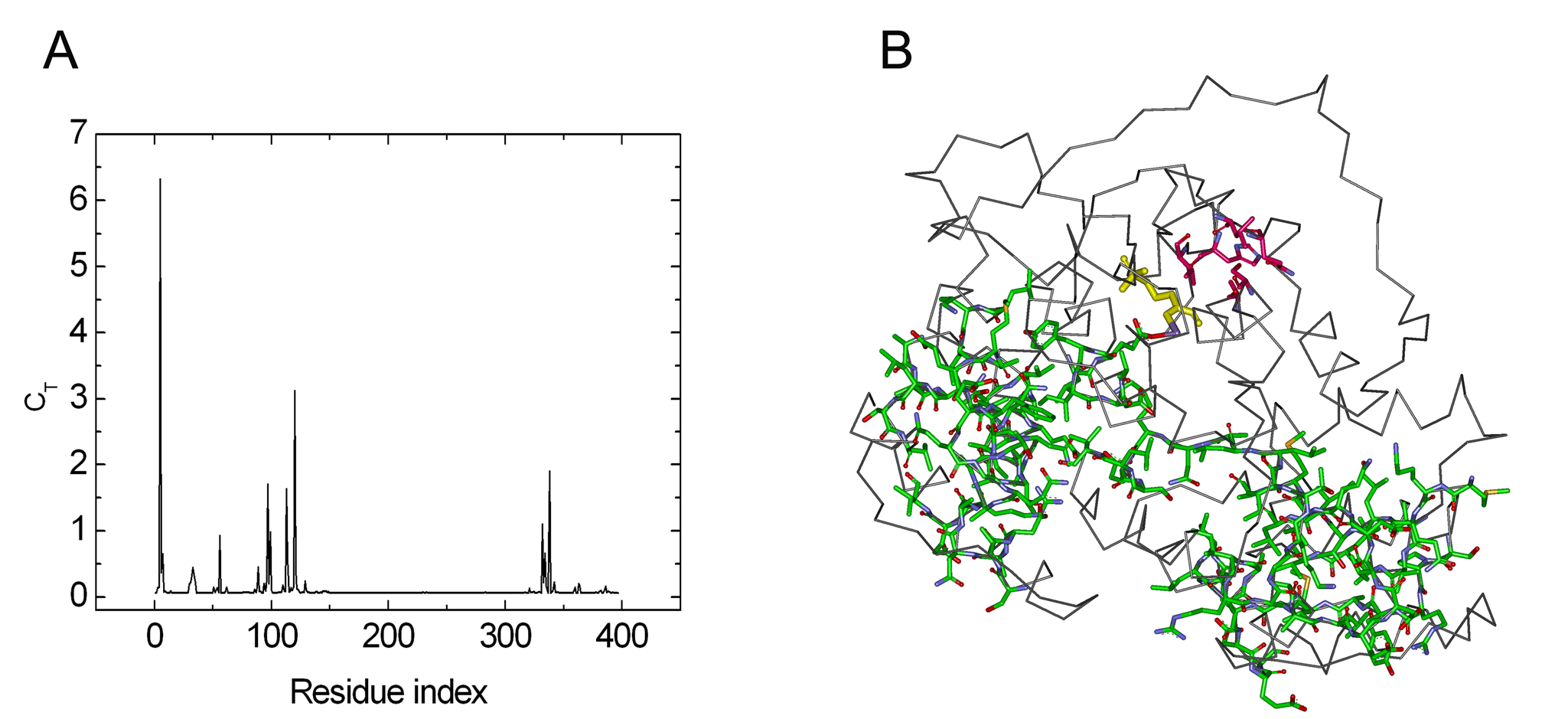
**

**Figure 2 Ispc**

1. **Transferases**

**
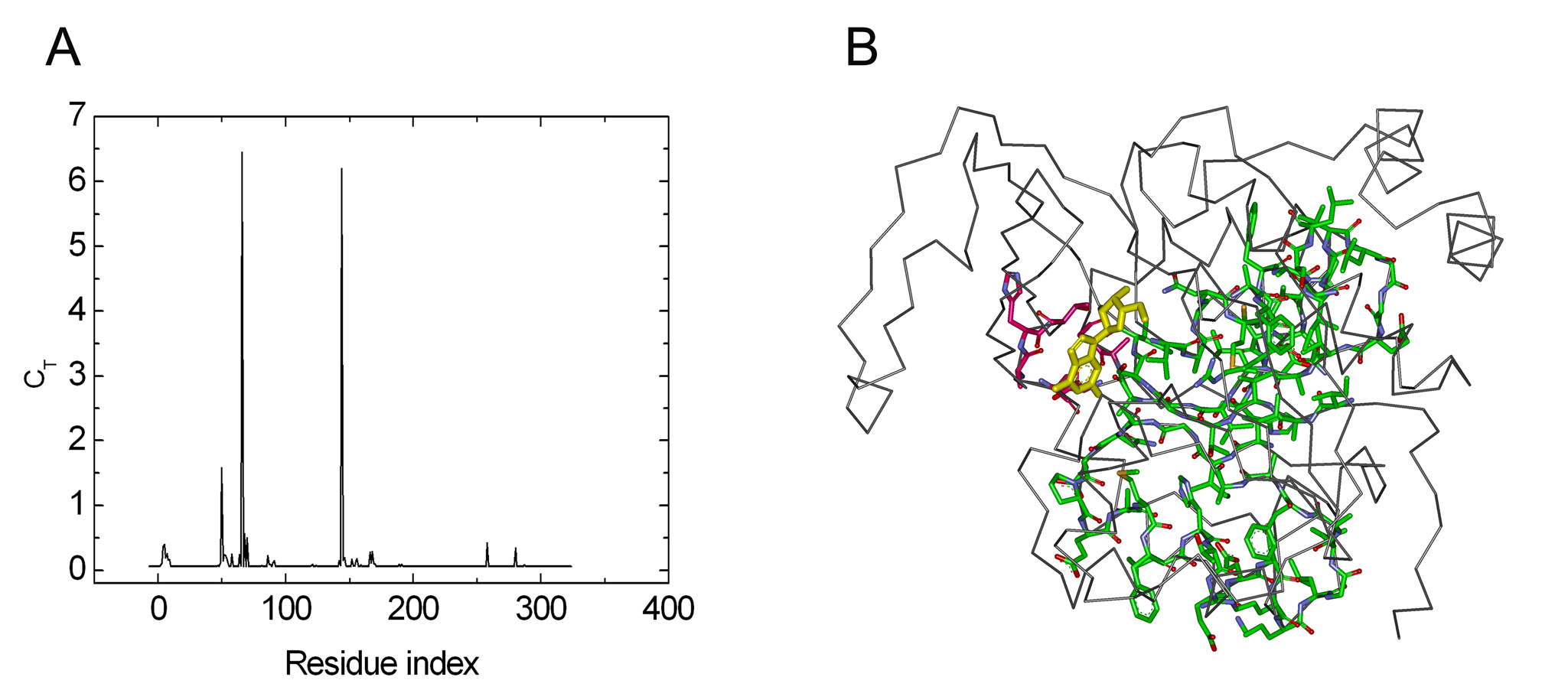
**

**Figure 3 Adenosine Kinase**

**
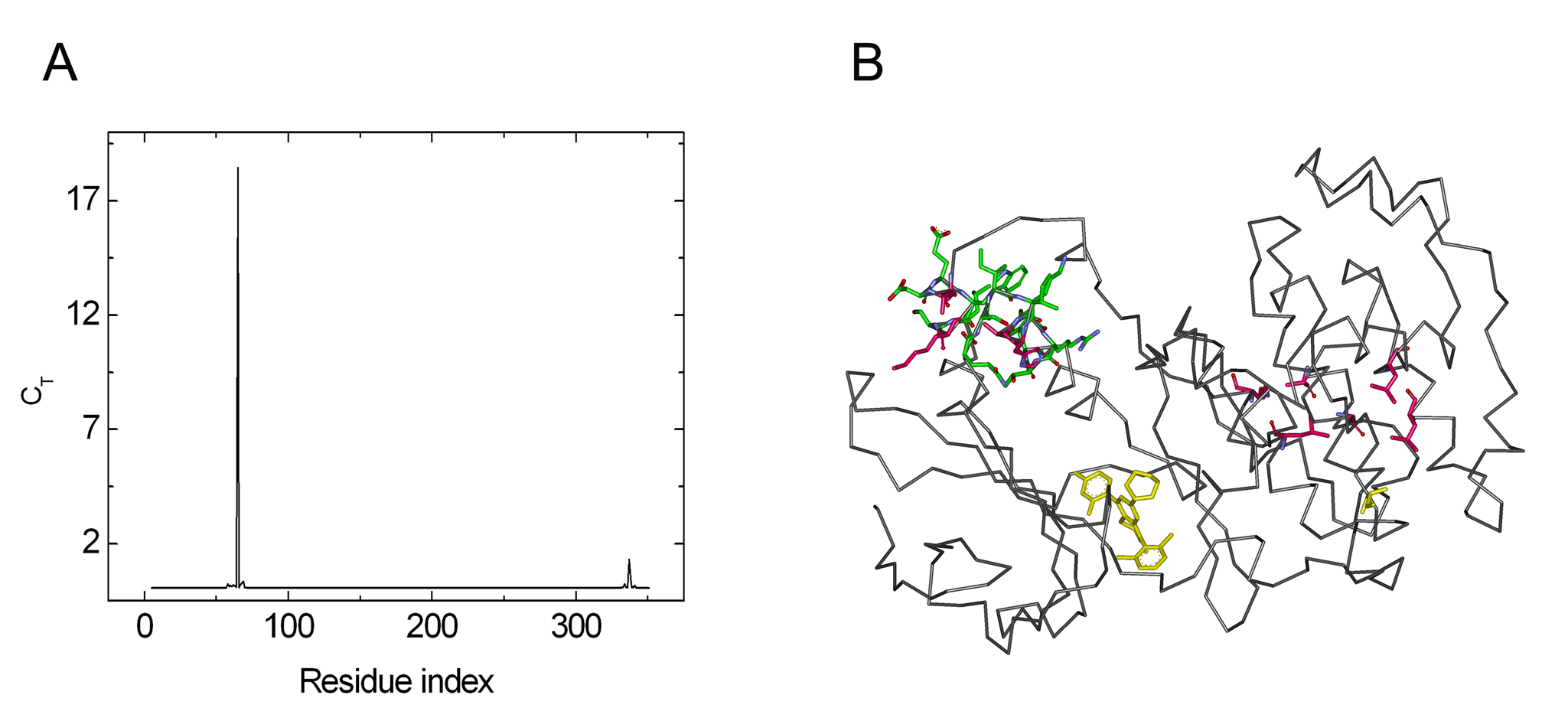
**

**Figure 4 Map Kinase P38-α**

**
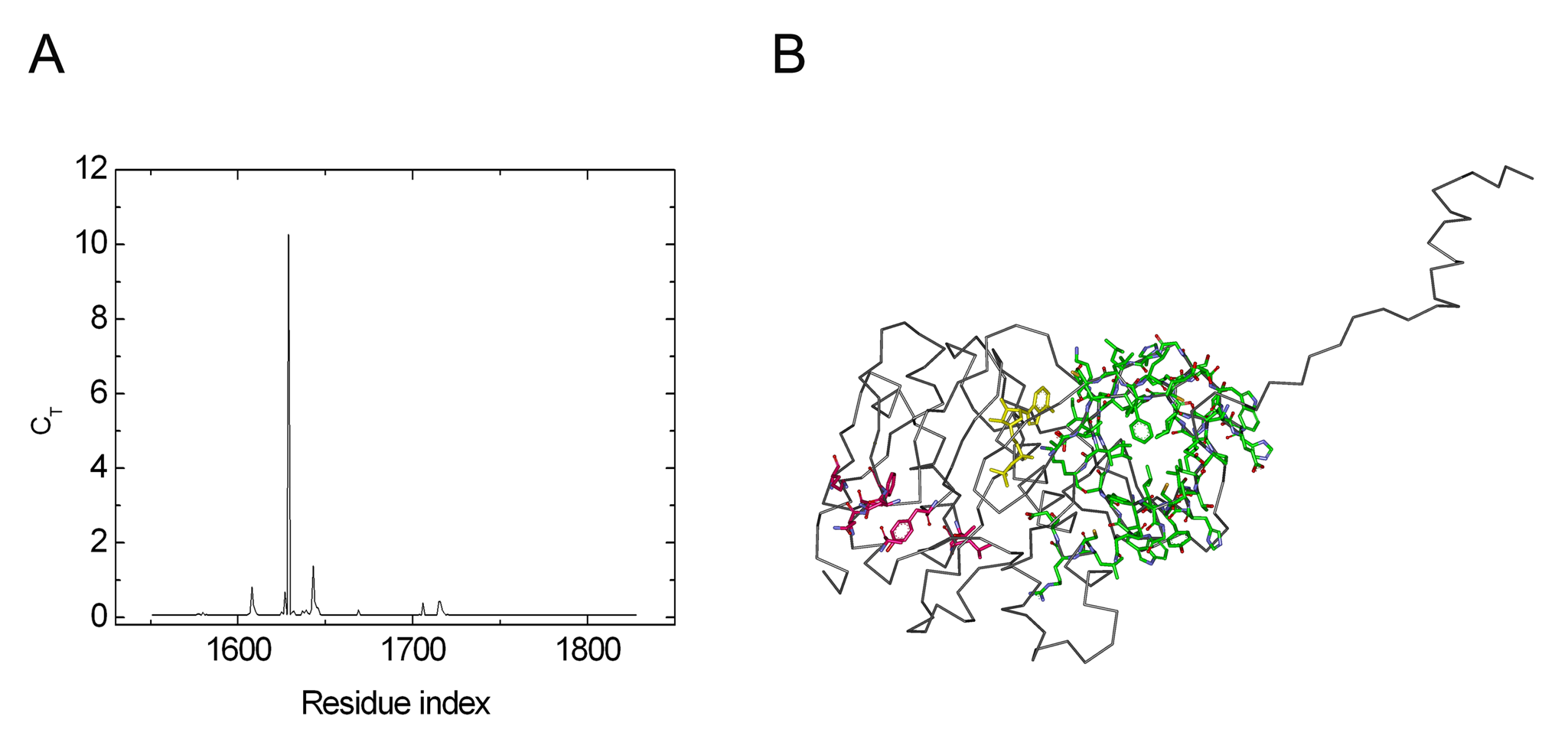
**

**Figure 5 Kinase Domain TRP-** **Ca^+2^** **Channel**

**
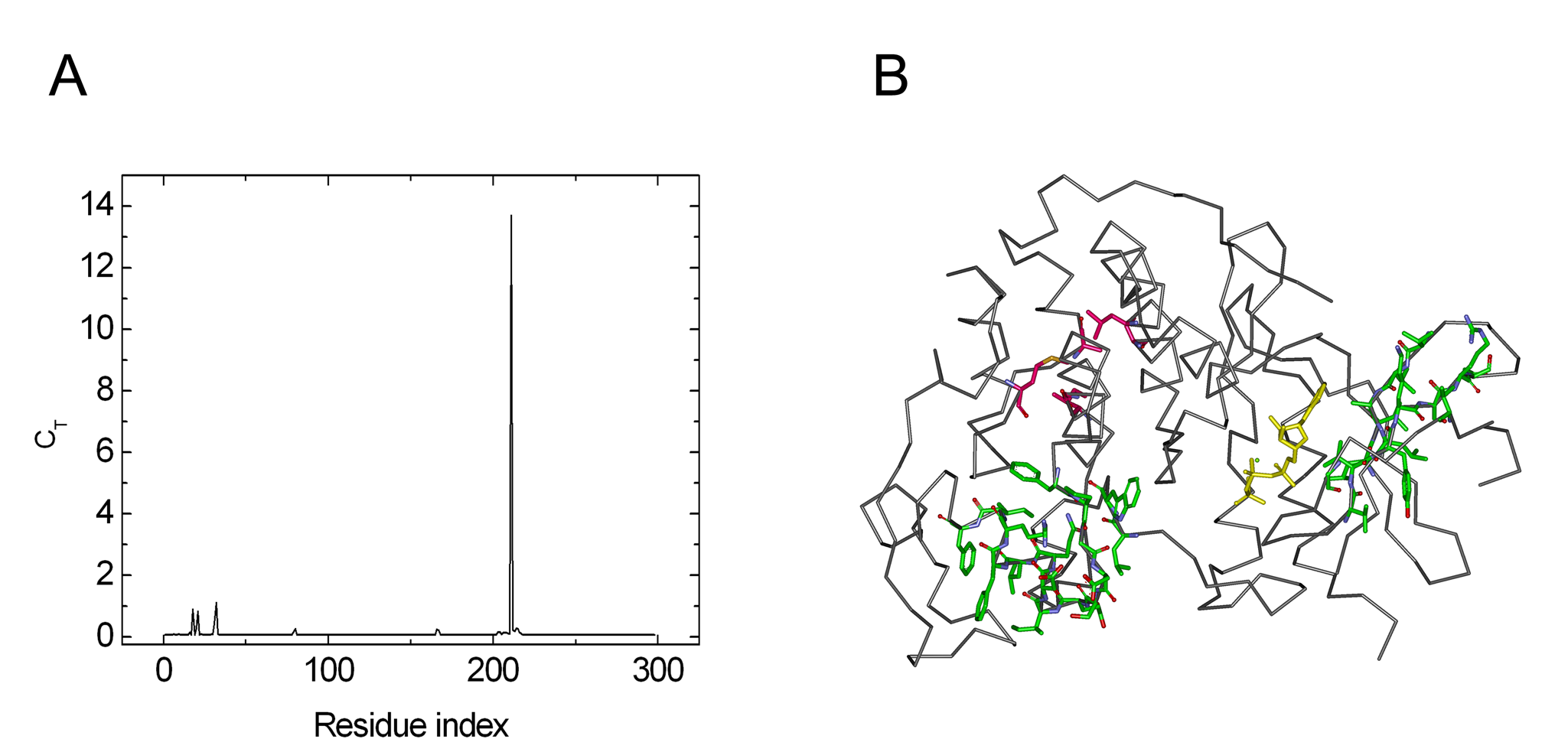
**

**Figure 6 Cyclin-Dependent Kinase**

**3. Hydrolases**

**
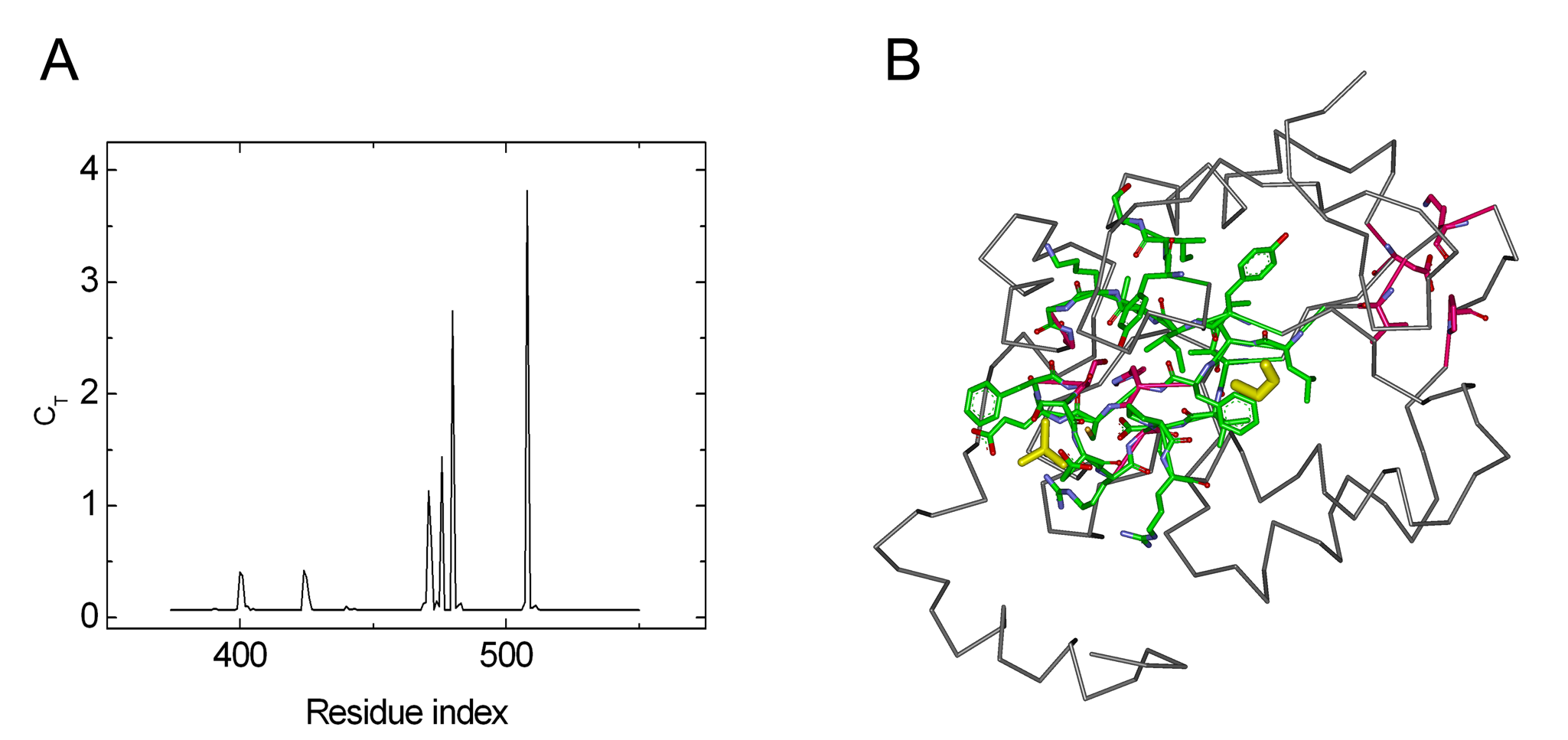
**

**Figure 7 M-phase inducer phosphatase 2 (Cdc25b)**

**
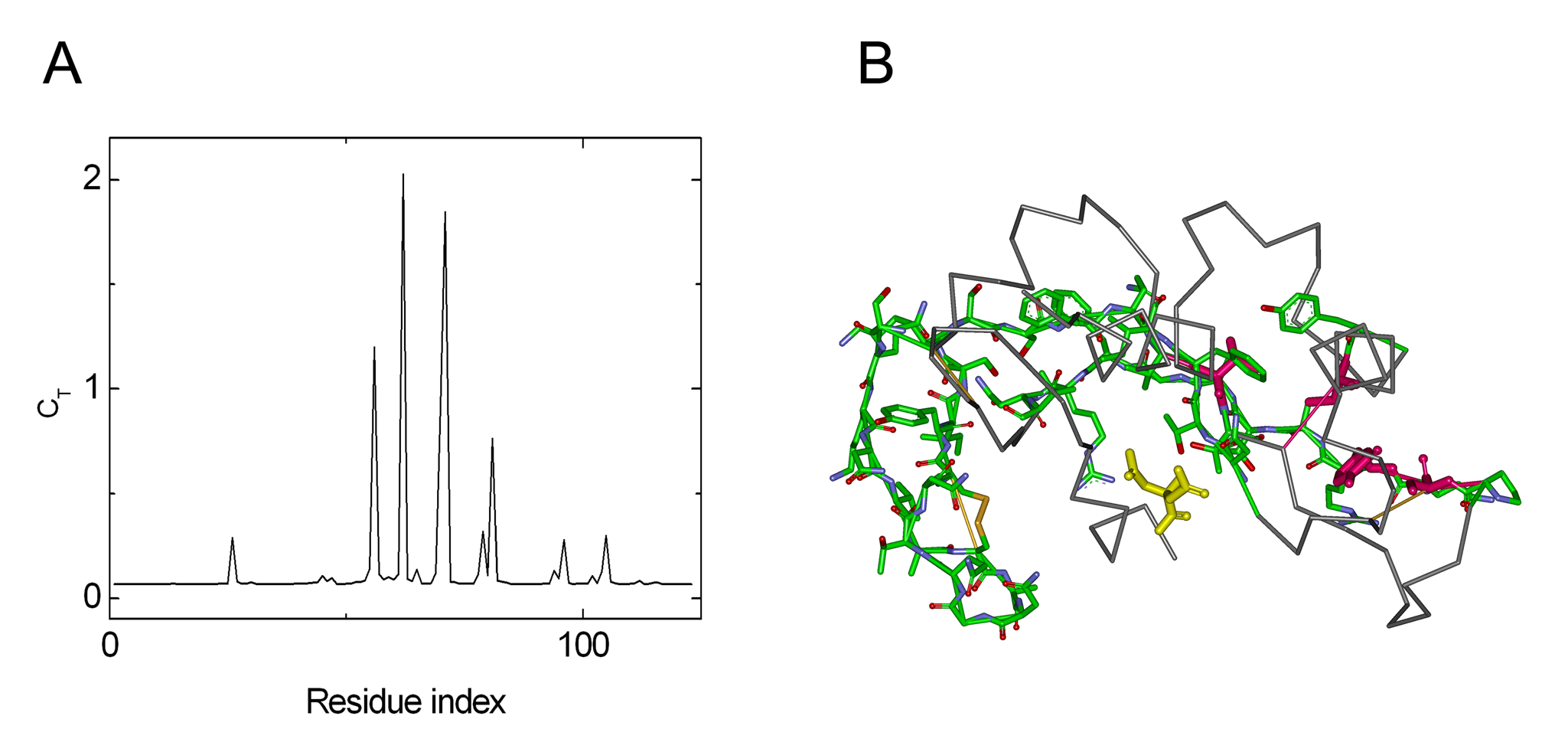
**

**Figure 8 Angiogenin**

**
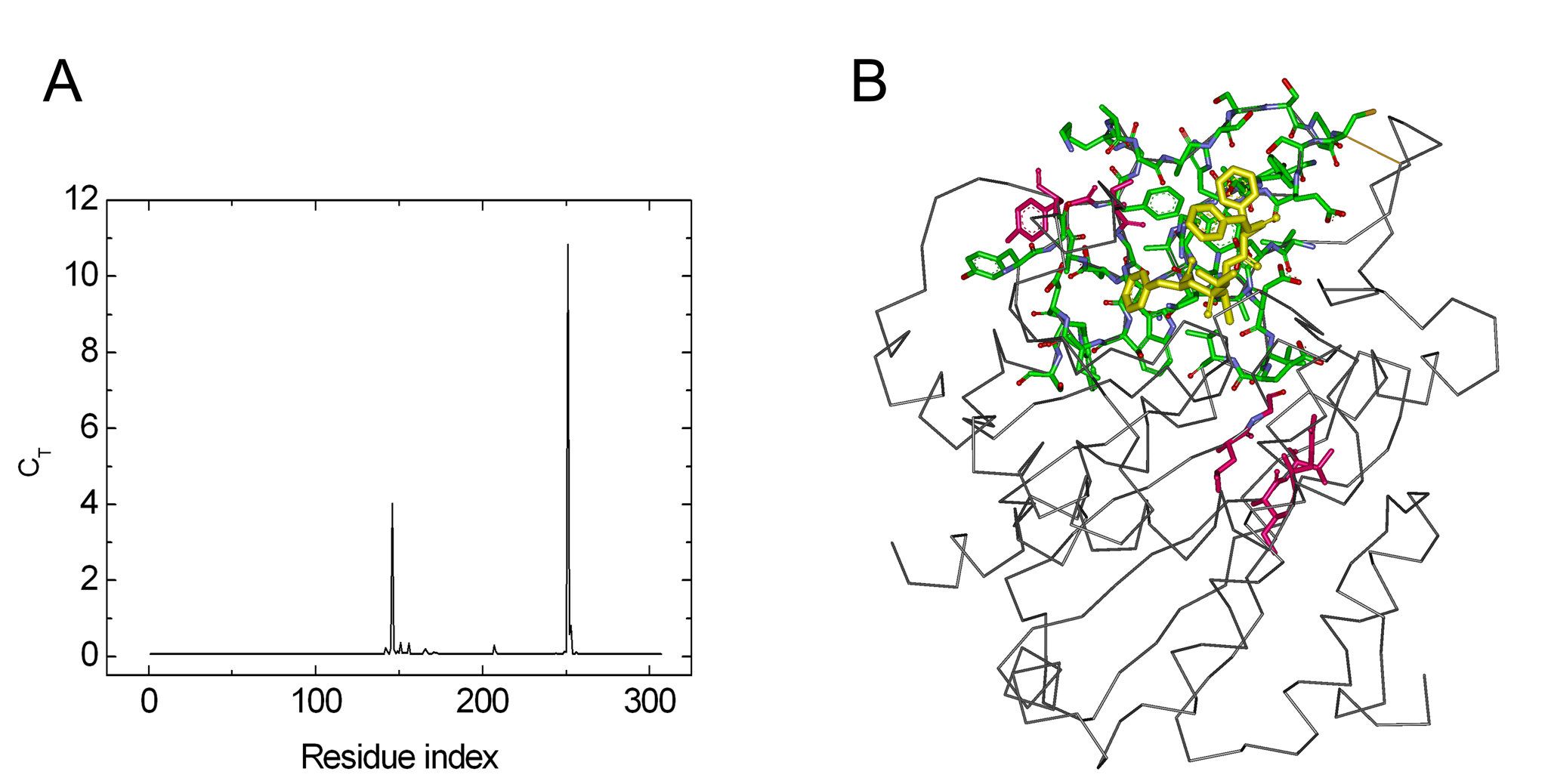
**

**Figure 9 Carboxypeptidase A**

**
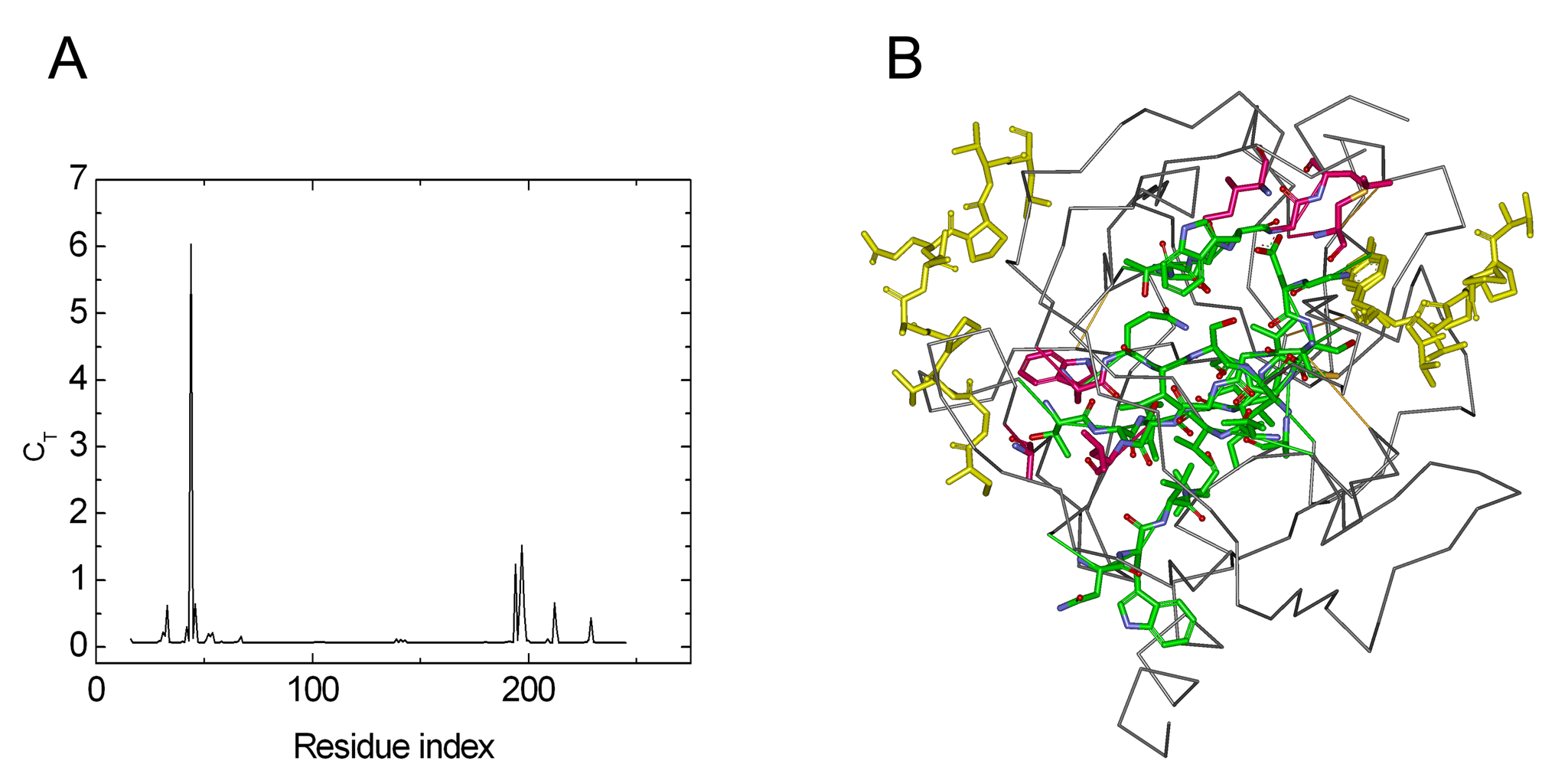
**

**Figure 10 Gamma Chymotyrpsin**

**
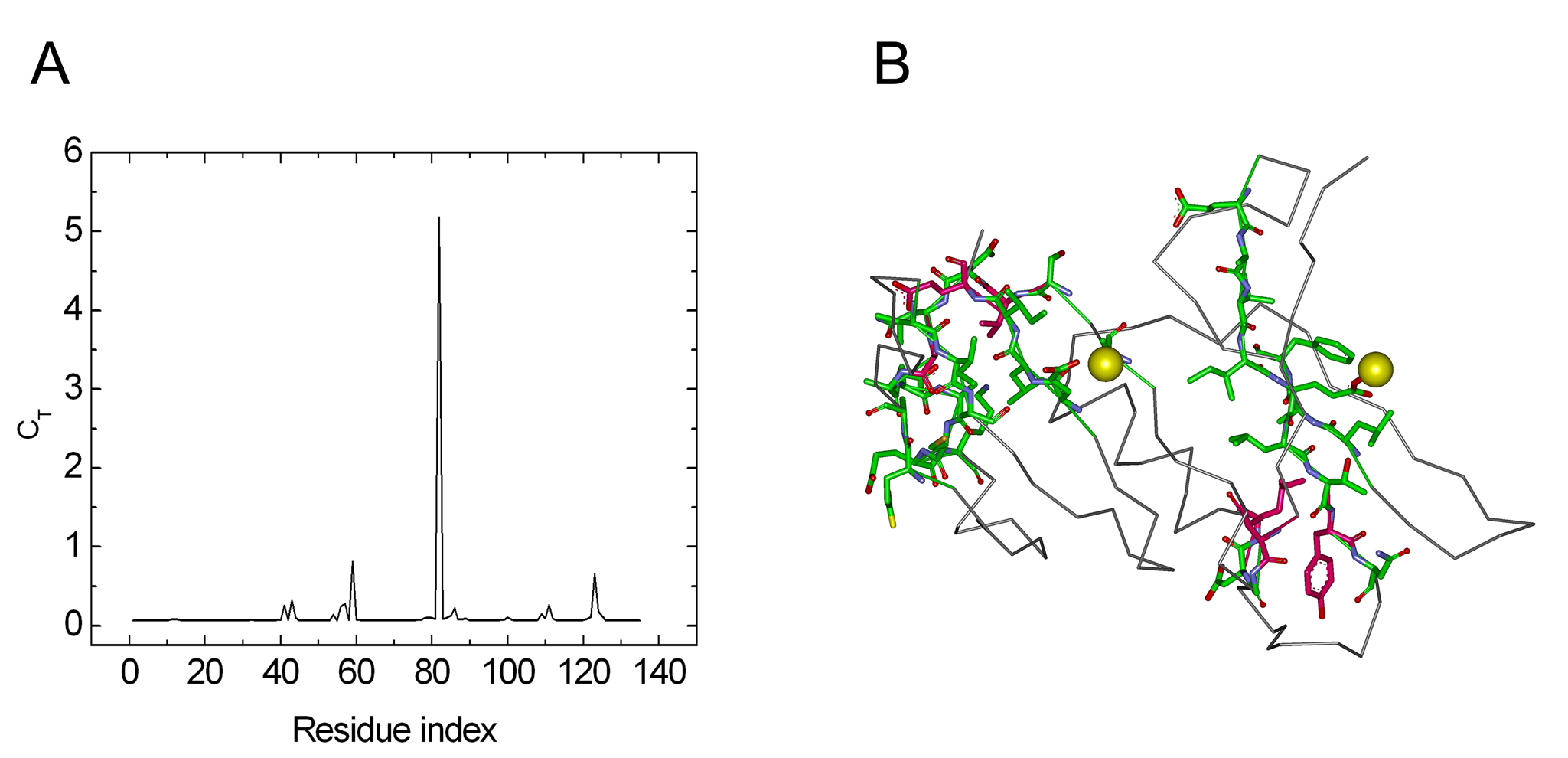
**

**Figure 11 Glyoxalase I**

**
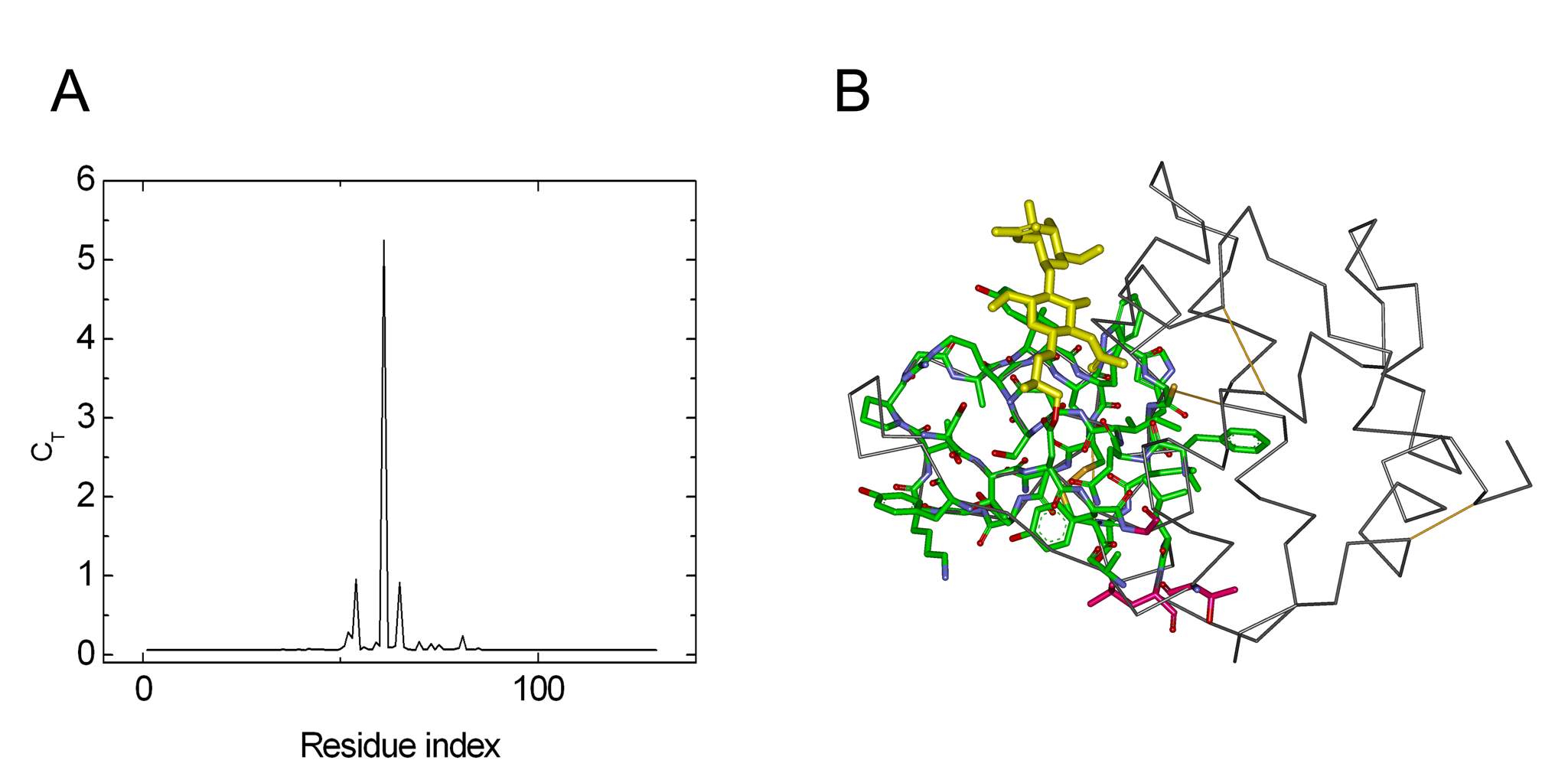
**

**Figure 12 Lysozyme**

**
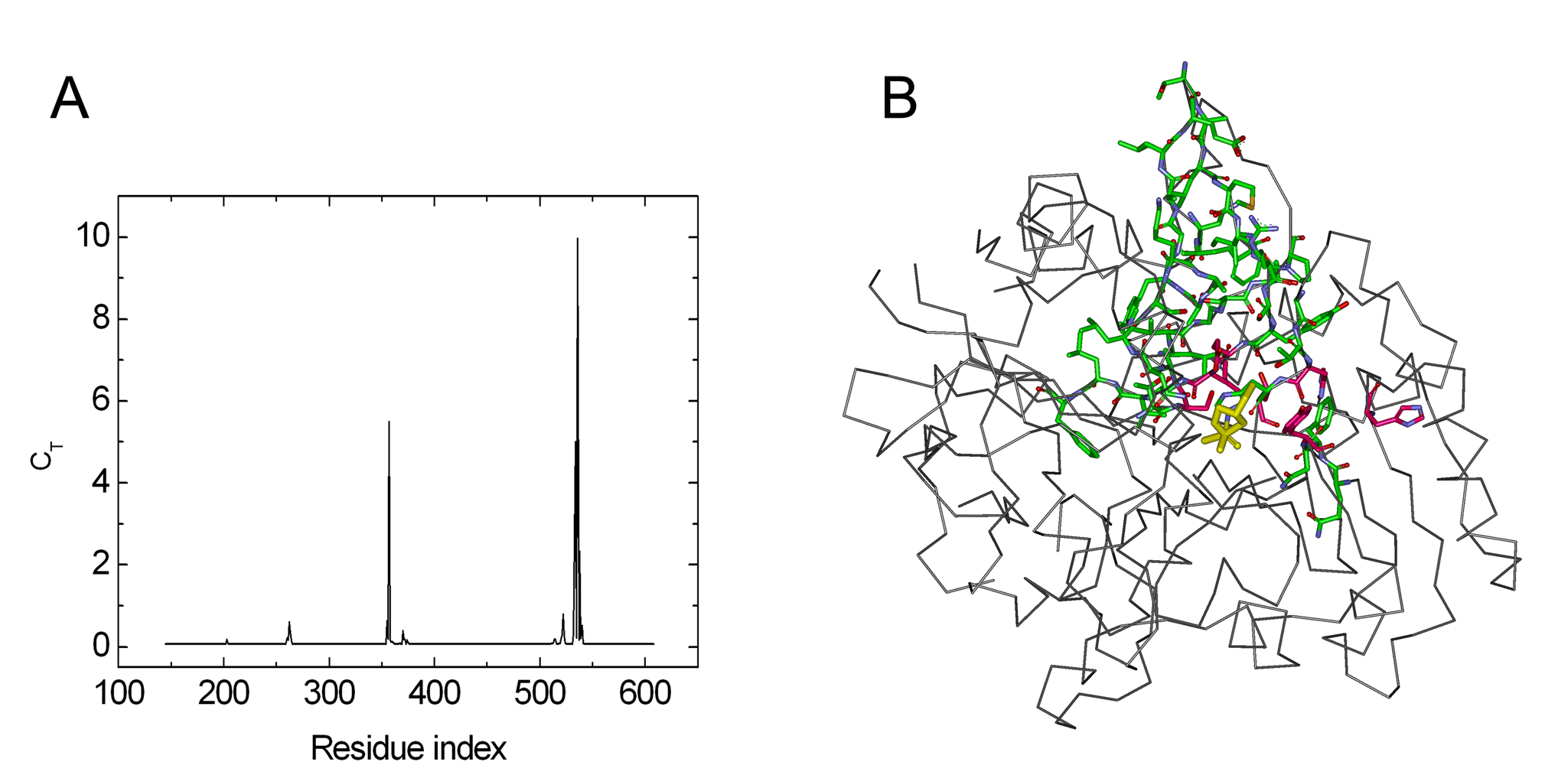
**

**Figure 13 Tyrosyl-DNA phosphodiesterase**

**
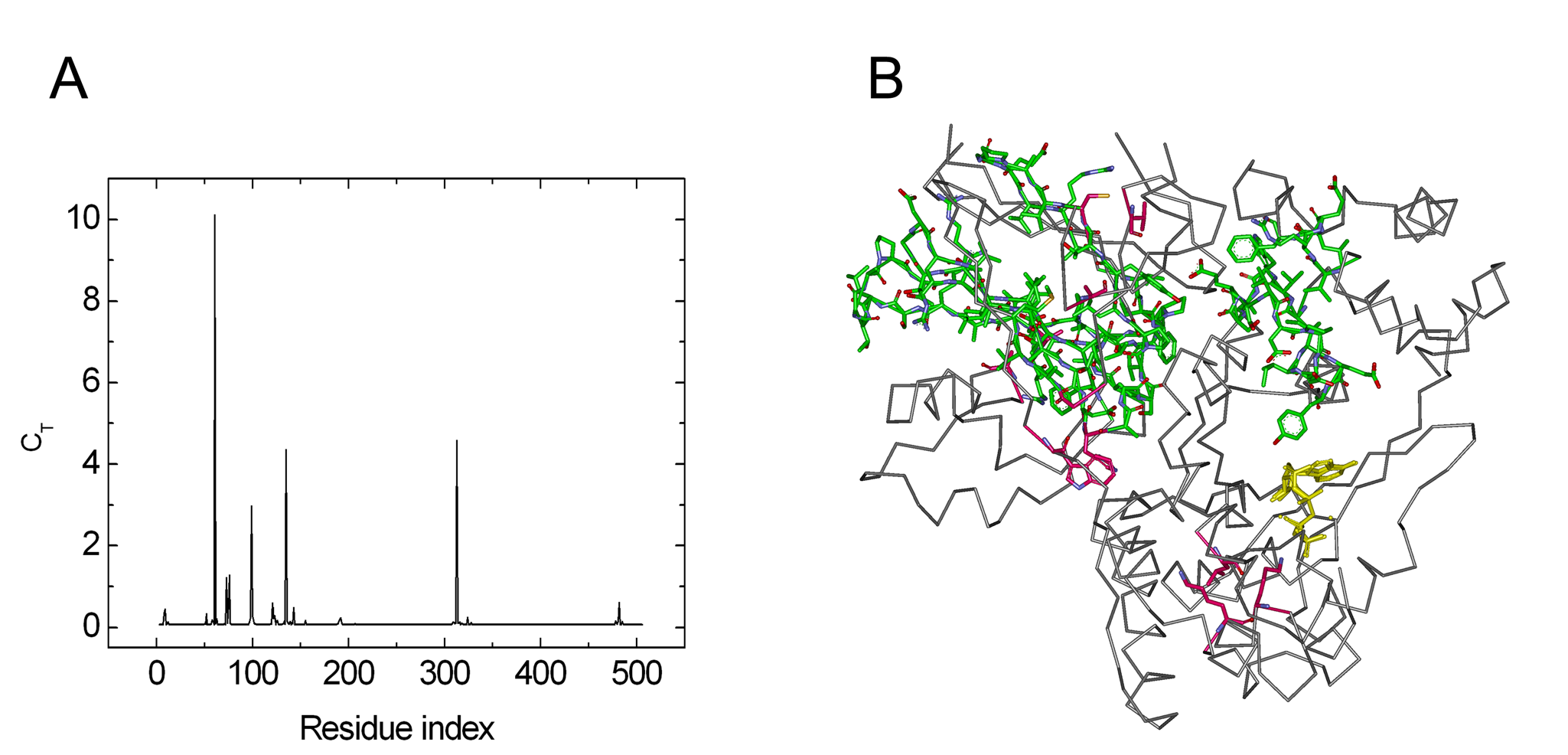
**

**Figure 14 Beta-lactam synthetase**

**
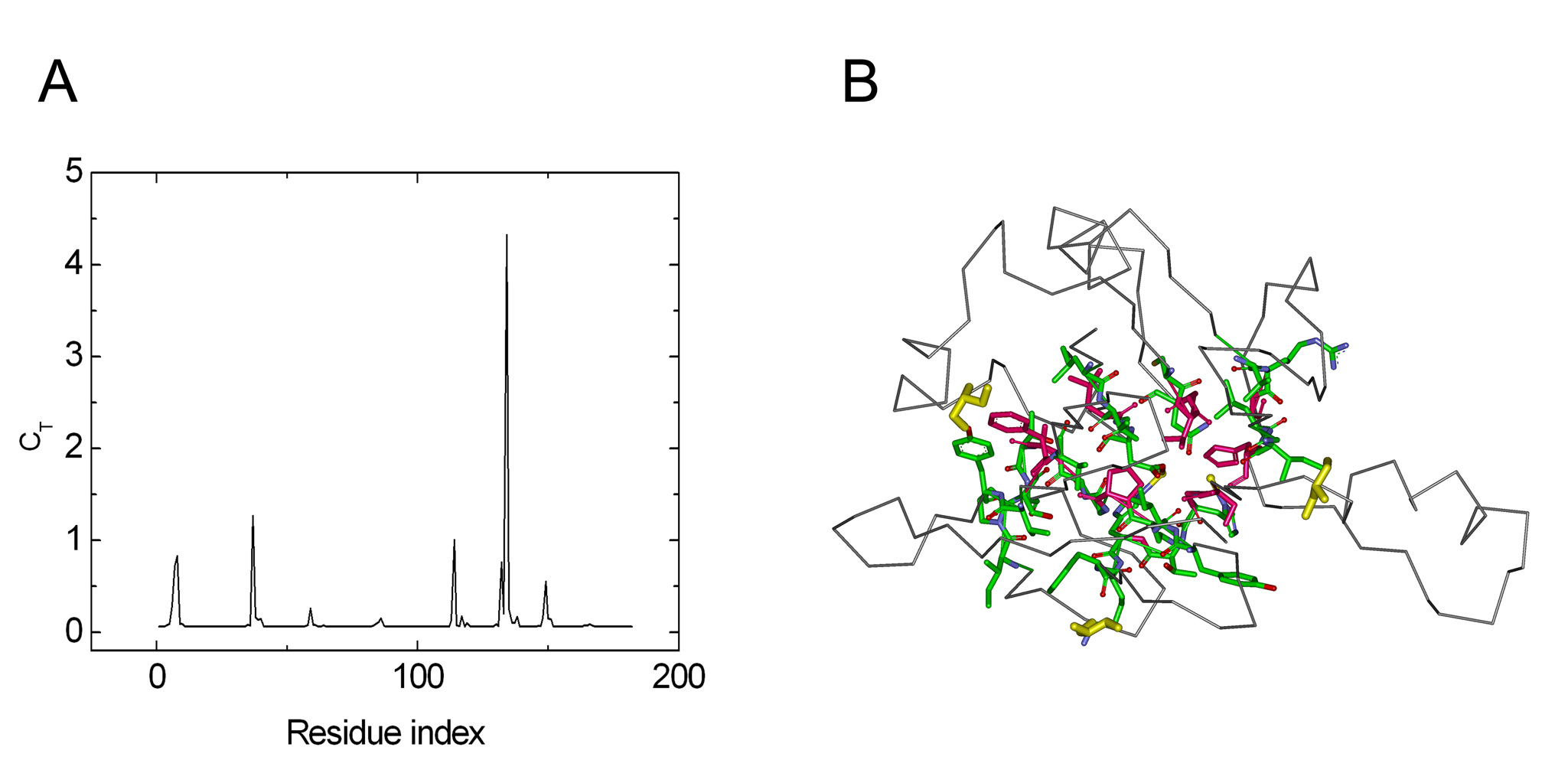
**

**Figure 15 Vacuolar protein sorting Protein29 (VPS29)**

**
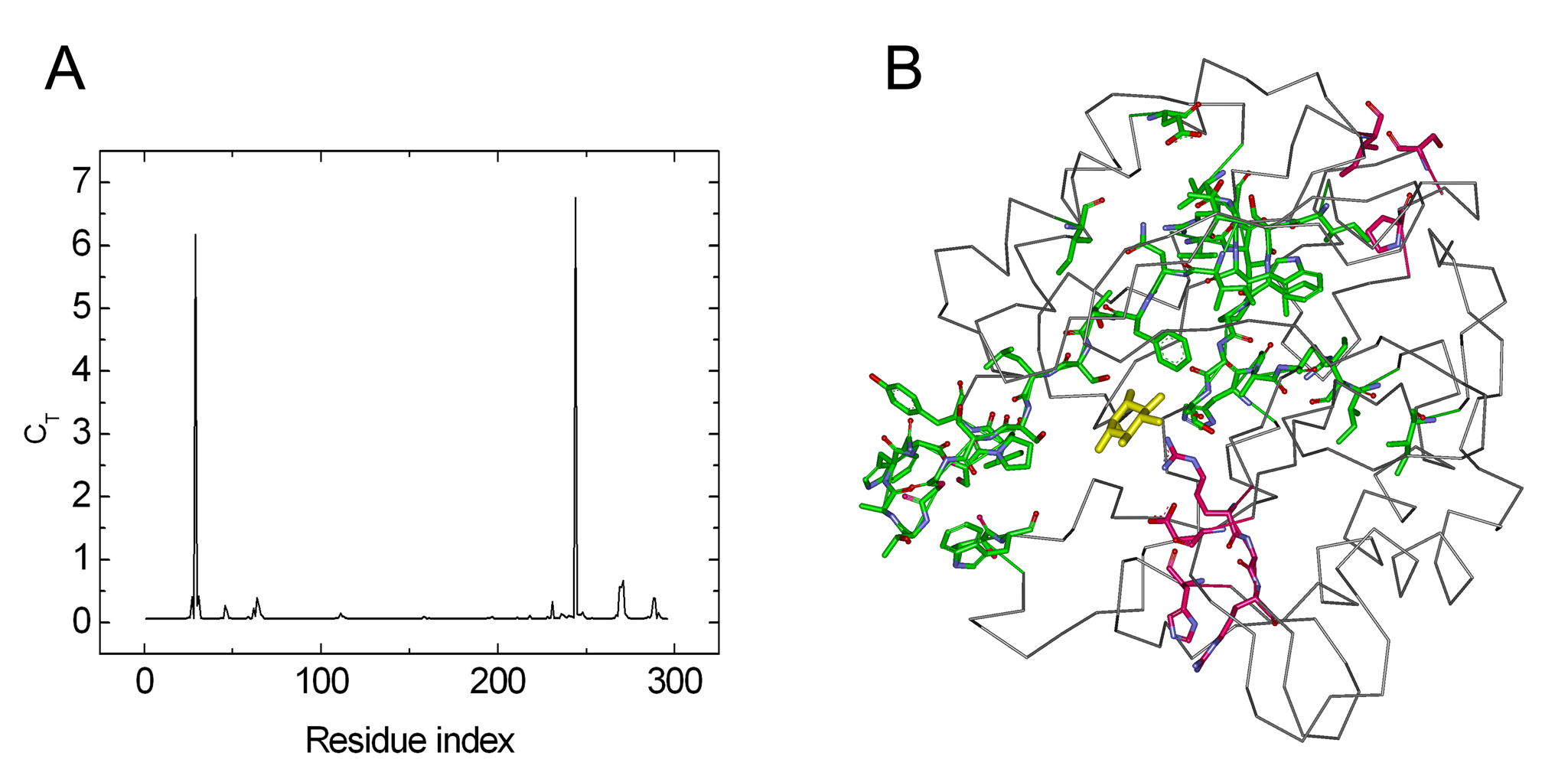
**

**Figure 16 Phospholipase C**

**
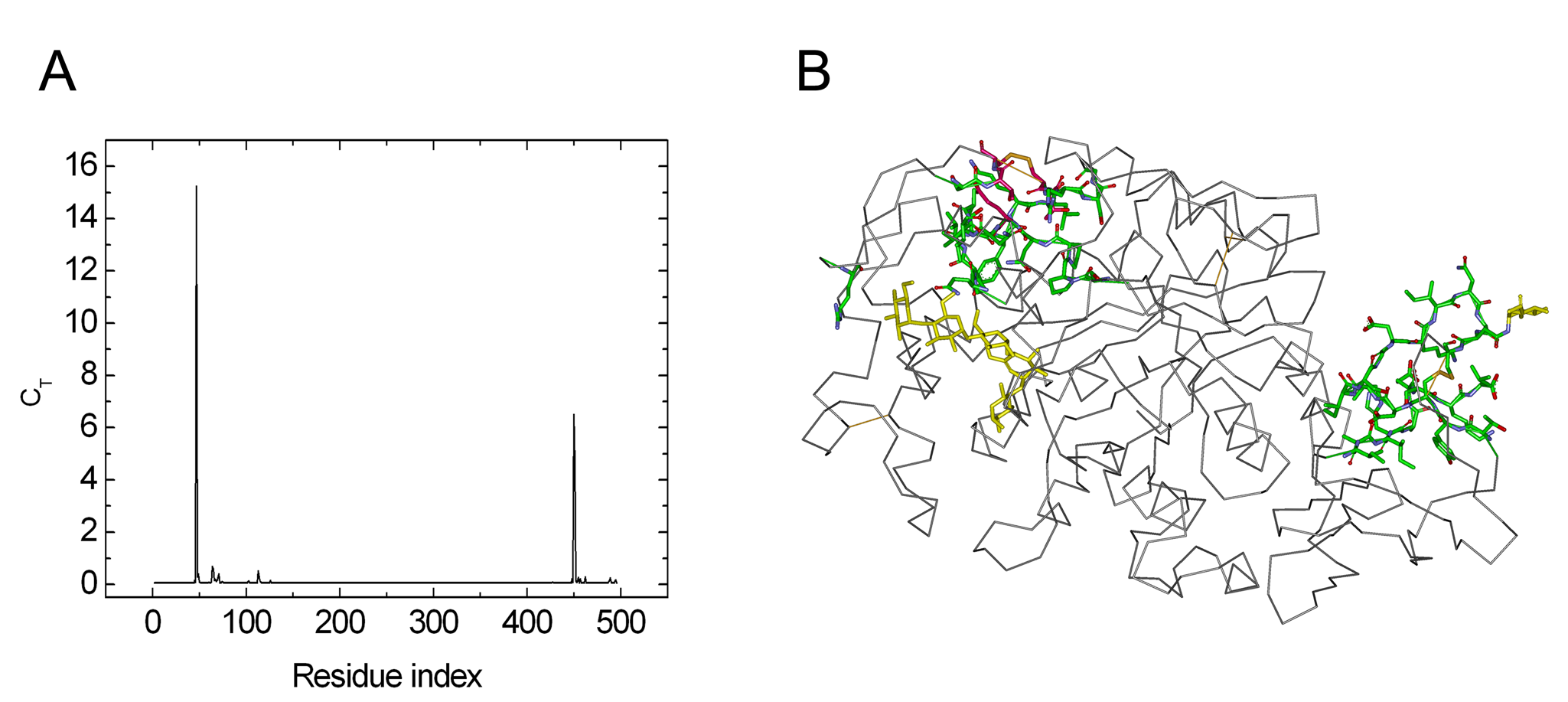
**

**Figure 17 Pancreatic α-amylase**

1. **Lyases**

**
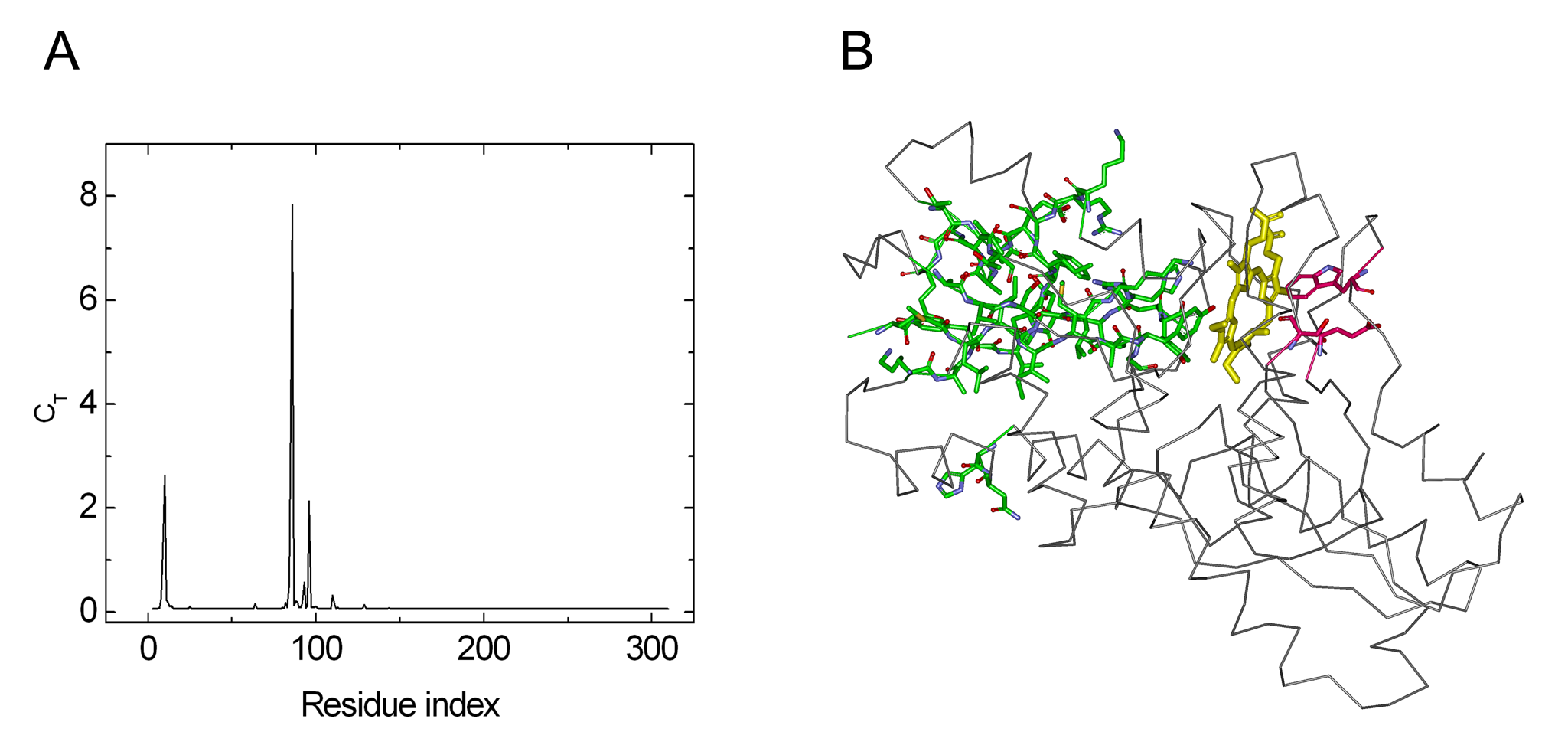
**

**Figure 18 Ferrochelatase**

**
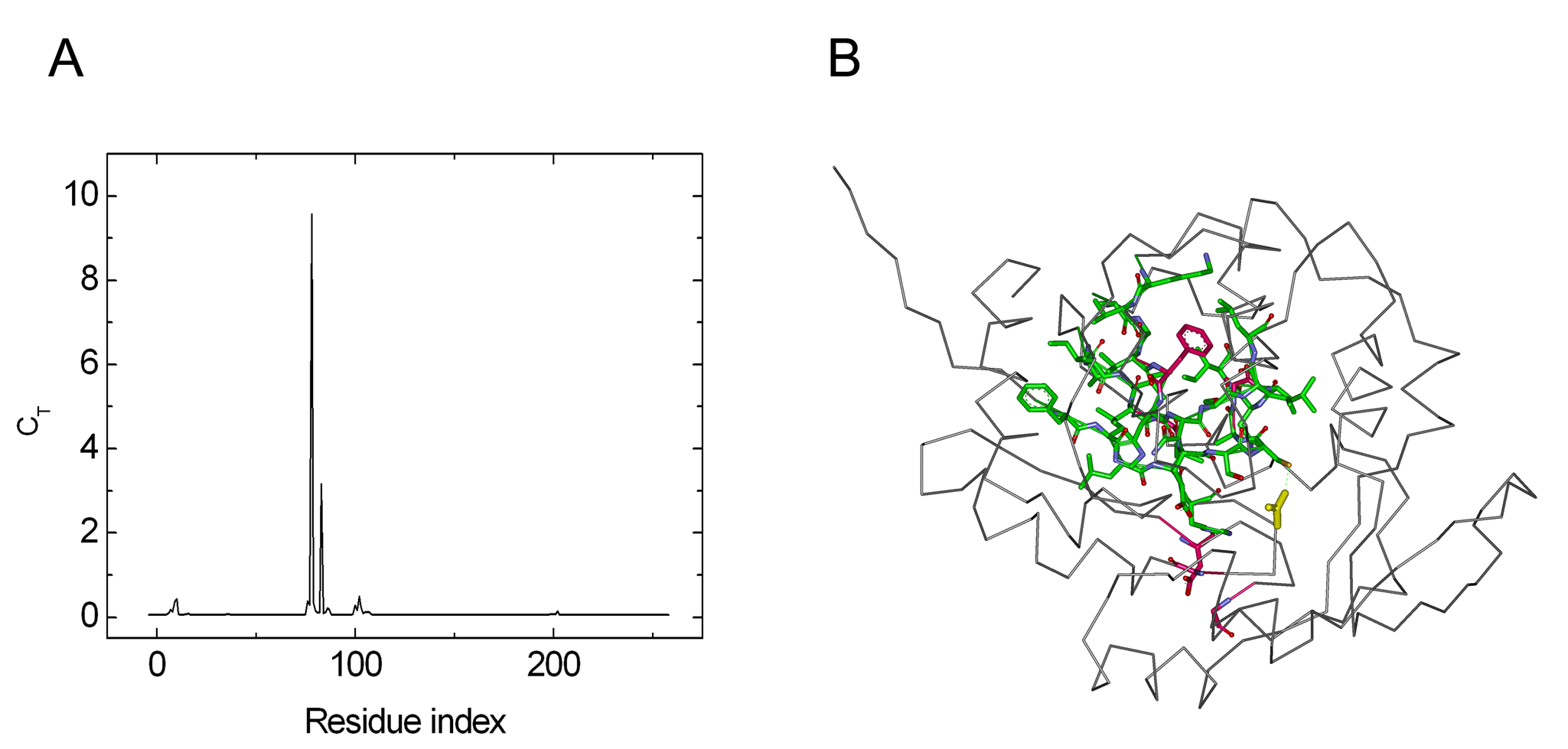
**

**Figure 19 Hydroxynitrile Lyase**

1. **Non-enzymes**

**
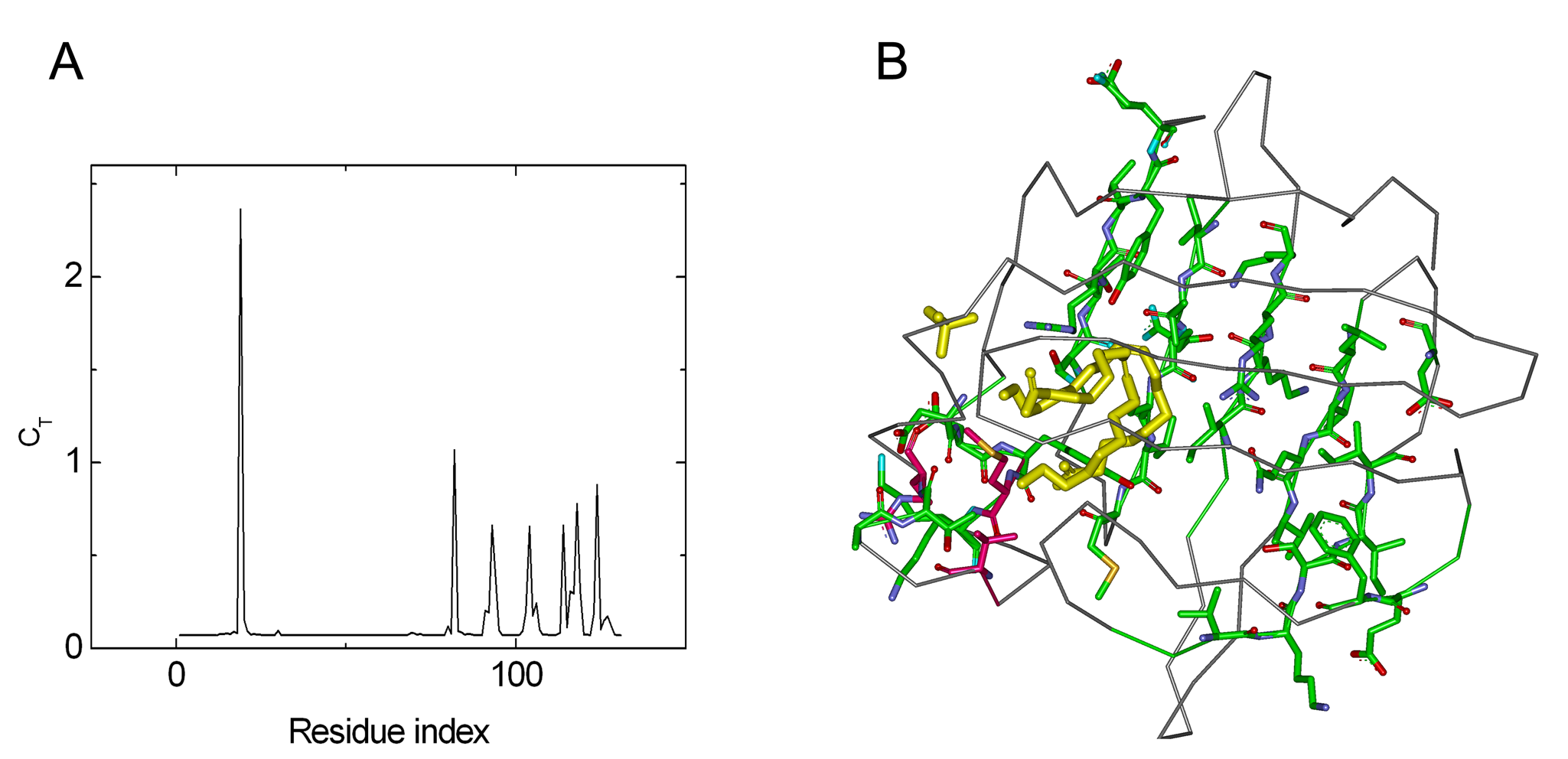
**

**Figure 20 Adipocyte Lipid binding Protein**

**
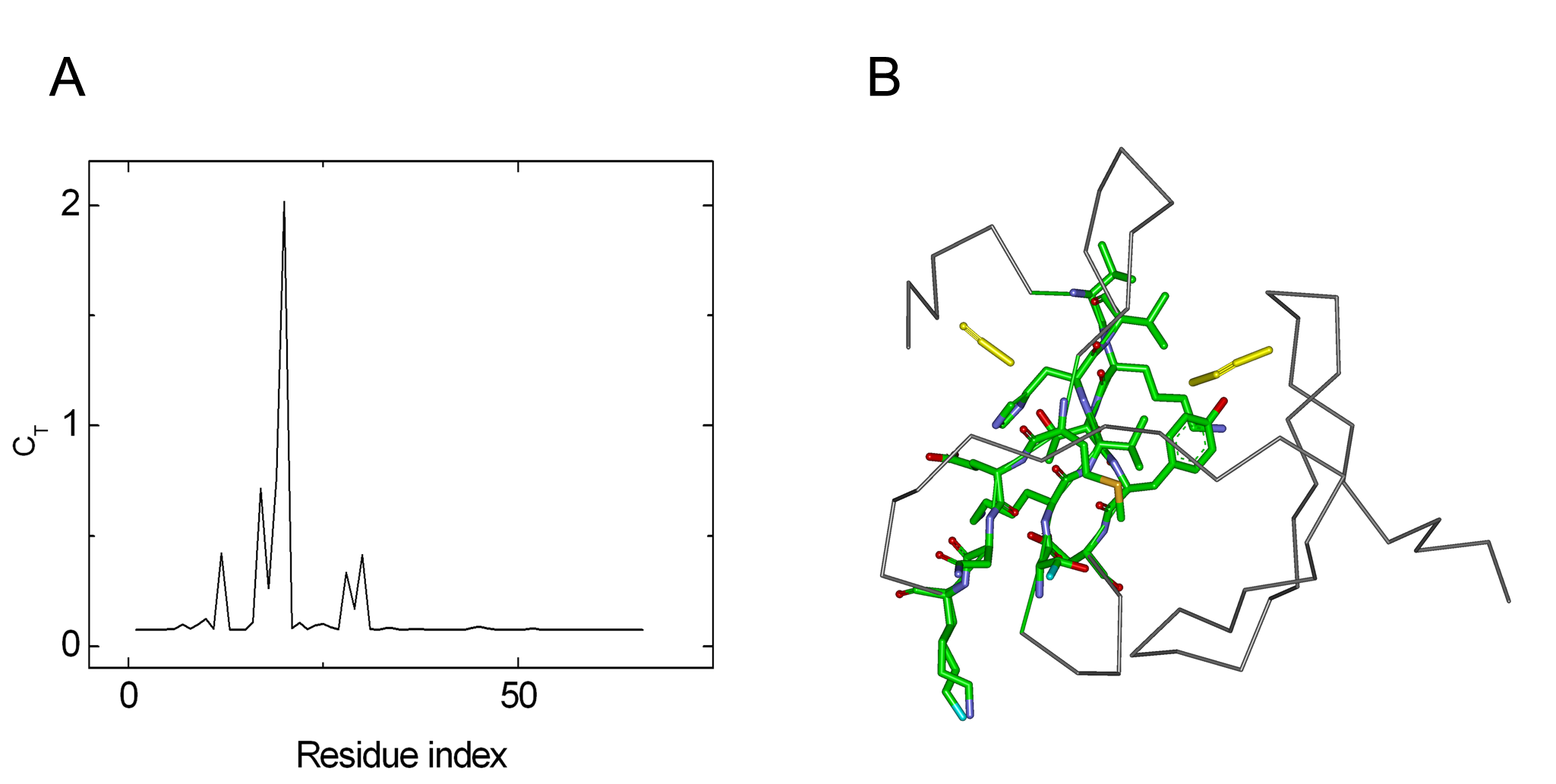
**

**Figure 21 Copper Resistance Protein**

**
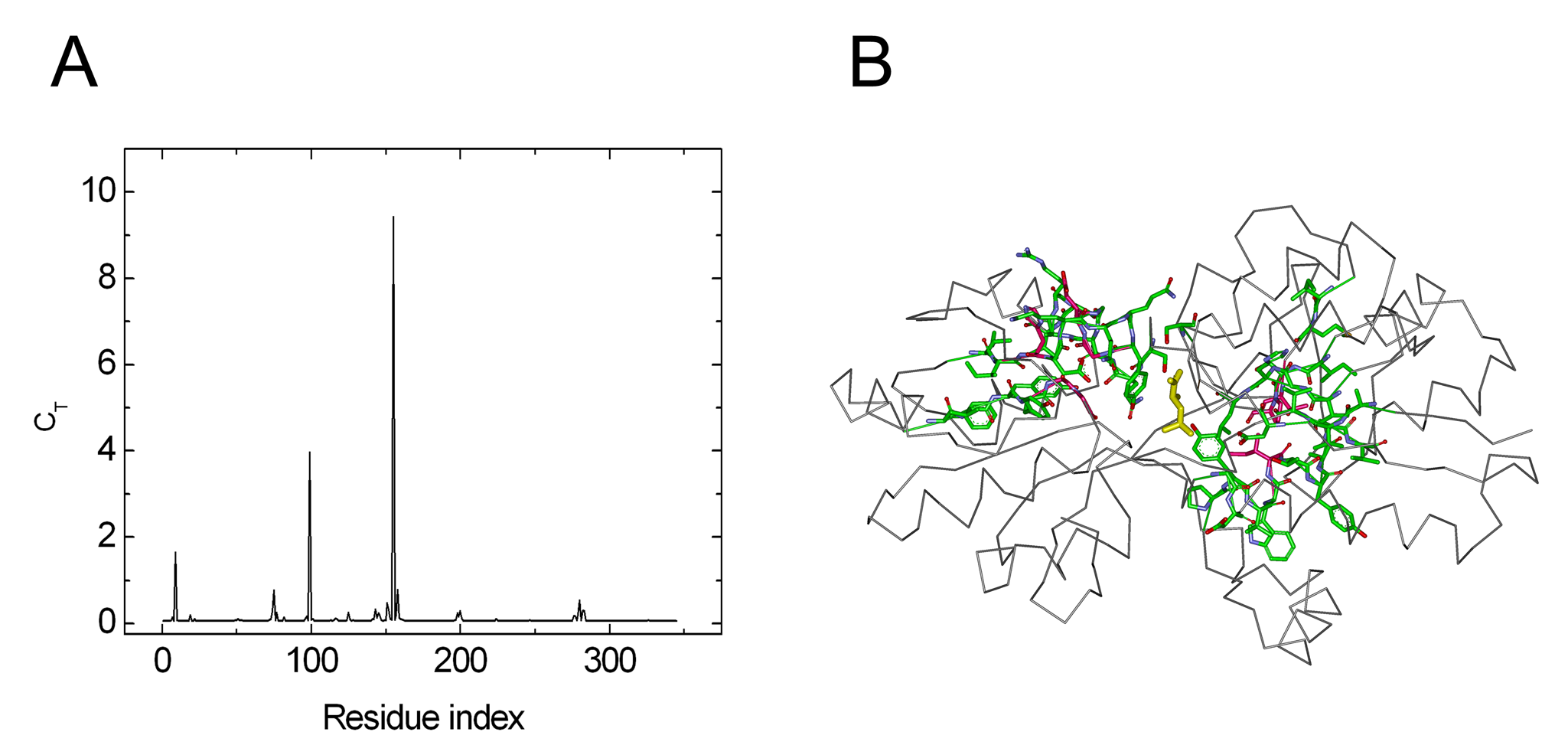
**

**Figure 22 L-leucine Binding Protein**

**
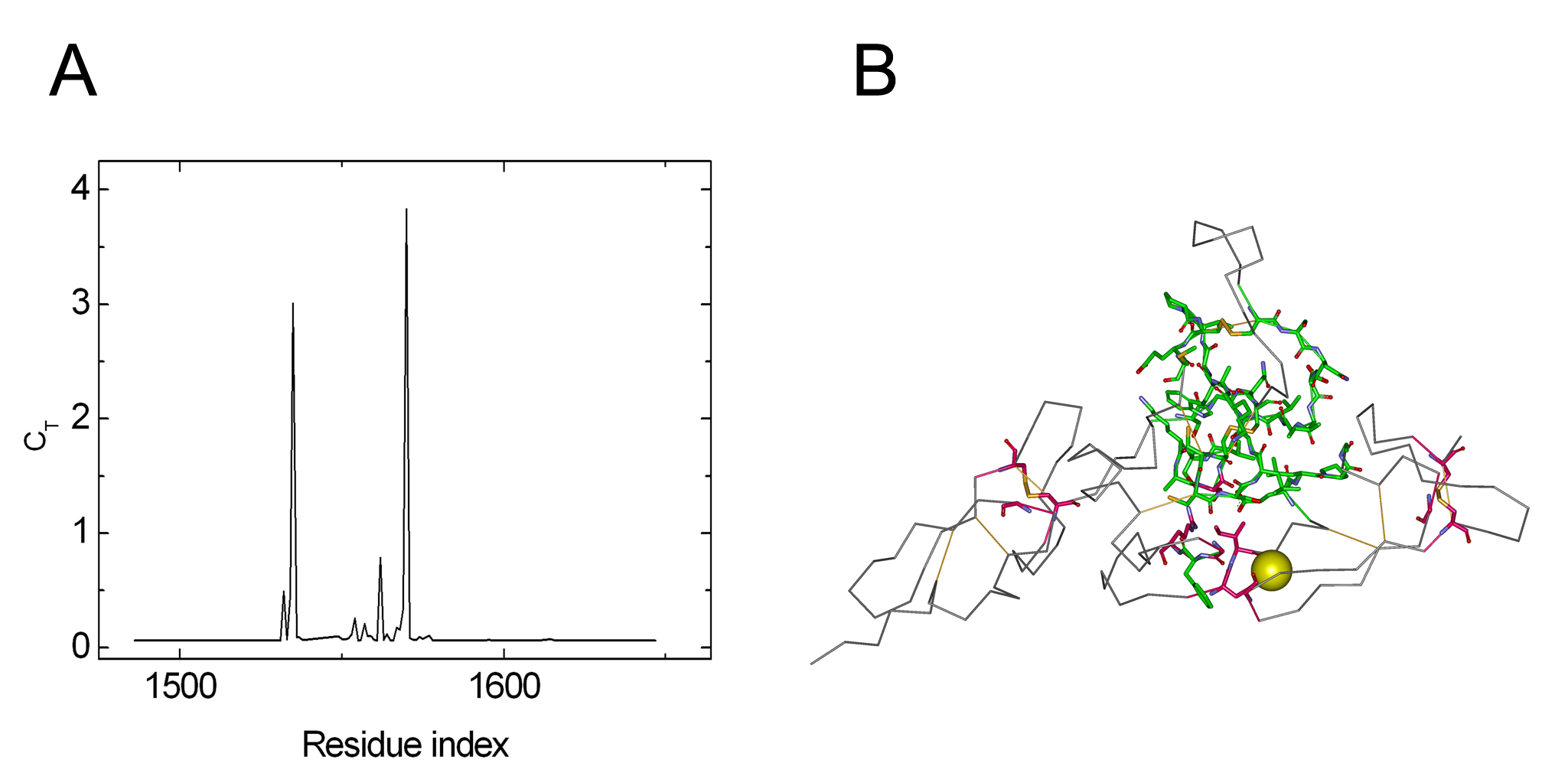
**

**Figure 23 Fibrillin-1**

**
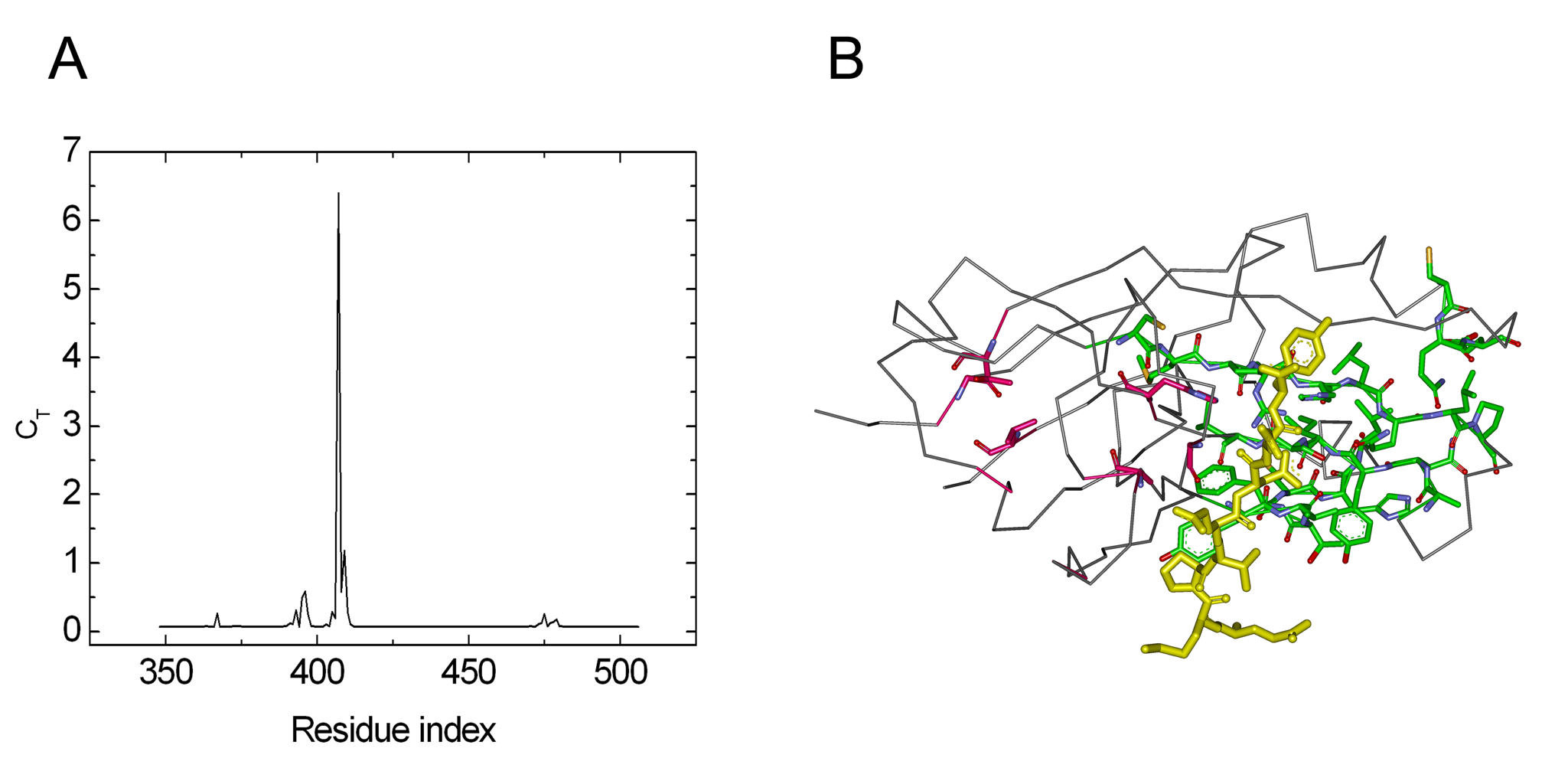
**

**Figure 24 TNF receptor associated factor (Traf 6)**
