# Supplementary material for: Identification of Ligand Binding Sites of Proteins Using the Gaussian Network Model
Source: PLoS One. 2011 Jan 25;6(1):e16474. doi: 10.1371/journal.pone.0016474 (PMC3026835; doi:10.1371/journal.pone.0016474)
Supplement: Supporting Information S2 — Log-Log plots of the relation between Number of residue-residue contact versus and of the relation between number, N, of cliques versus . (DOCX) [file pone.0016474.s002.docx]

SCALING OF NUMBER OF RESIDUE CONTACTS WITH CUTOFF DISTANCE,


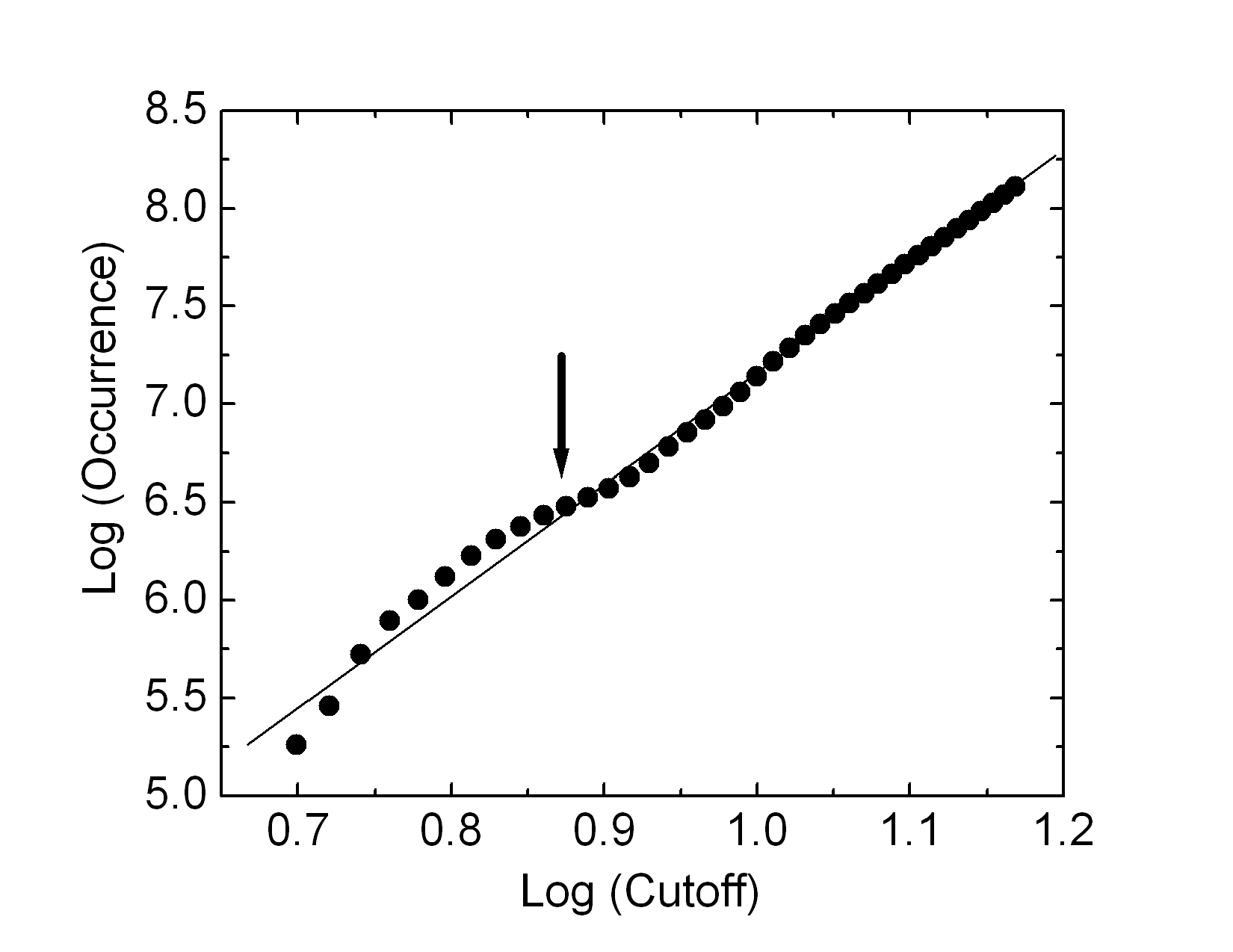


Figure 1. Log-Log plot of the relation between Number of residue-residue contact versus .
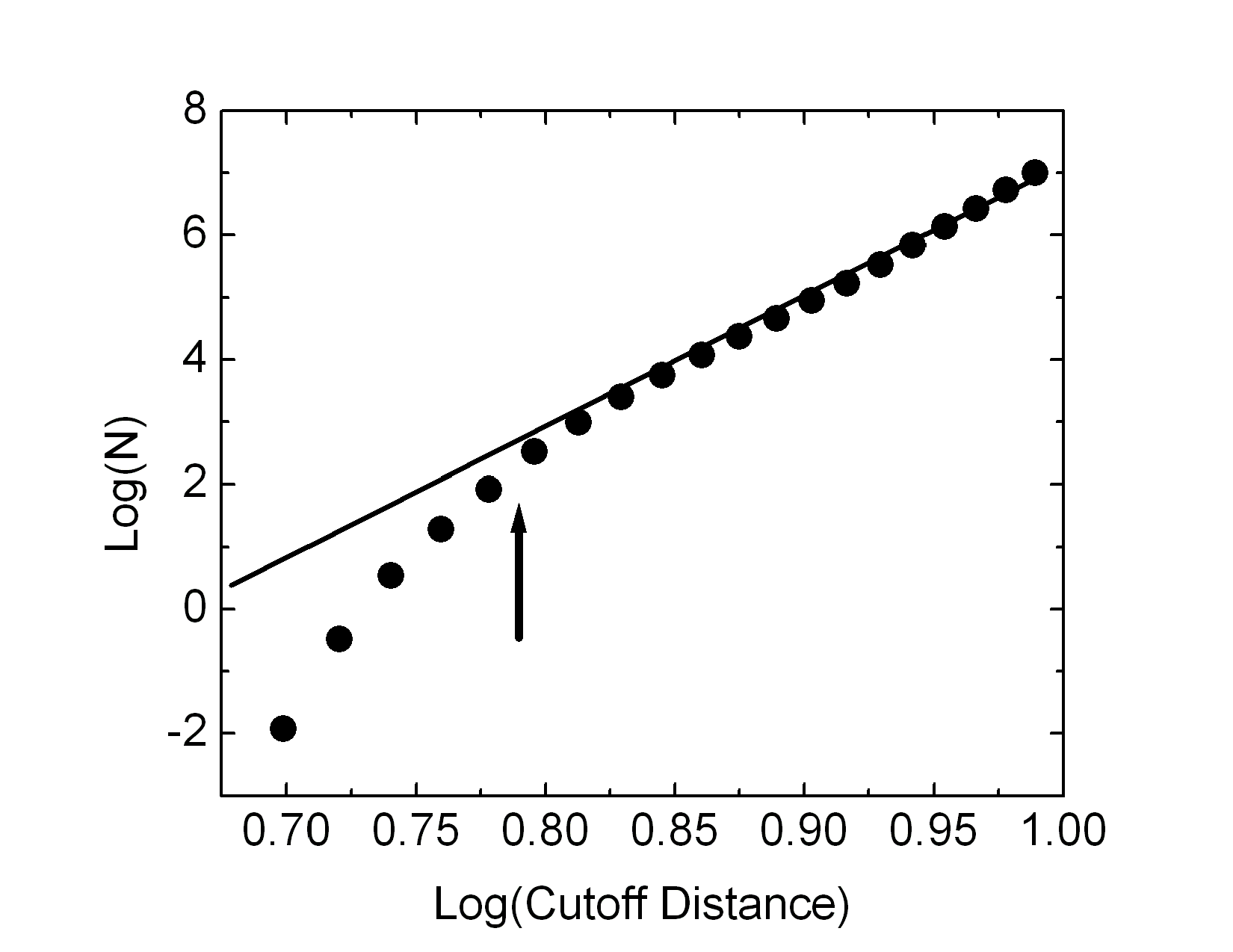


Figure 2. Log-Log plot of the relation between number, N, of cliques versus .
